# Diversity and Plasticity of Hydraulic Traits Across a Relative Moisture Supply Gradient: Results From Multiple Common Gardens

**DOI:** 10.1111/pce.70890

**Published:** 2026-09-16

**Authors:** Duncan D. Smith, Thomas J. Givnish, Mark A. Adams, Katherine A. McCulloh

**Affiliations:** ^1^ Department of Botany University of Wisconsin – Madison Madison Wisconsin USA; ^2^ School of Ecosystem and Forest Sciences, University of Melbourne Creswick Victoria Australia; ^3^ School of Life and Environmental Sciences, University of Sydney Sydney New South Wales Australia

**Keywords:** climate gradient, common garden, *Eucalyptus*, hydraulic conductivity, plasticity, turgor loss point, xylem anatomy, xylem density

## Abstract

Maintaining cellular hydration is essential for plant metabolic activity and carbon gain. Plants typically maintain hydration through efficiency of water transport networks, the ability to avoid network disruption, and retention of water via accumulation of osmotica. Given their global domain and their enduring presence across geological eras, plants have evolved with large variations in moisture supply across geographic and temporal space, notably via trait adaptation and plasticity. We quantified plastic and genetically fixed variation in 6 key hydraulic traits across 10 *Eucalyptus* species in 4 common gardens spanning a climatic relative‐moisture gradient in Victoria, Australia. These traits generally shift plastically within species across gardens while maintaining interspecific trends across species within gardens. Species grown at or originating from drier sites tend to have lower hydraulic conductivity, narrower xylem conduits, greater conduit density, greater bulk xylem density, greater fibre wall fraction, and more negative turgor loss point, consistent with expectations based on maximizing whole‐plant growth. Genetically fixed variation was usually greater than plastic variation, but some traits were more plastic in species native to moister areas. Variation in xylem anatomy largely explained variation in hydraulic conductivity and xylem density, notwithstanding idiosyncratic site and species effects.

## Summary Statement

In 10 *Eucalyptus* species grown across 4 common gardens spanning a climate gradient, 6 hydraulic traits shifted plastically with garden climate, varied with species' climates of origin, and were correlated with one another to help explain species' distributions and adaptation to climate variation.

## 1 Introduction

Carbon and water are major currencies in the adaptive economy of plants (Givnish and Vermeij 1976; Cowan and Farquhar 1977; Ehleringer and Werk 1986; Givnish 1986a, 1986b; McCulloh et al. 2003, 2011; Sperry et al. 2017; Smith et al. 2023). Carbon, acquired by assimilation of atmospheric CO_2_ taken in through stomatal pores, is a crucial building block for growth, respiration, reproduction, and other processes. Water is necessary for carbon acquisition and metabolism because cellular machinery requires hydration to function properly. And yet, when stomata are open for CO_2_ intake, underlying cells will nearly always lose water to a comparatively dry atmosphere. Photosynthetic capacity of mesophyll tissue declines in species‐specific ways as leaf water potential declines (Tezara et al. 1999; Salvi et al. 2021). Lost water must be replaced if cellular hydration and carbon acquisition are to be maintained. Thus, water transport efficiency (hydraulic conductance) from soil to stomata strongly influences carbon gain (Hubbard et al. 2001). In vascular plants, hydraulic conductance is a complex function of xylem conduit diameters and abundance, transport distances and related properties. Hydraulic conductivity (conductance standardized by length and area) generally increases with wider conduit diameters and fewer conduits per area, owing to space‐filling constraints (McCulloh et al. 2011). Cellular hydration and function are also supported by other plant attributes. Stomata can partly or fully close, reducing water loss while maintaining cellular hydration, but at the expense of carbon intake. Symplastic solutes can act as osmotica, helping to retain water in cells (Kishor et al. 1995). Accumulated solutes can lower a cell's turgor loss point (TLP), aiding cellular growth (Steppe et al. 2018) and preventing subcellular disruption (Cheng et al. 2017). Some specific ‘compatible’ solutes, including amino acids such as proline and cyclitols such as quercitol, help stabilize proteins and maintain their enzymatic function even when cellular water content is low (Papageorgiou and Murata 1995; Jaindl and Popp 2006; Bozorgmehr and Monhemi 2015). Cellular water content can regulate the synthesis of such solutes (Arndt et al. 2008; Merchant et al. 2010; Ashraf et al. 2018).

Photosynthesis relies on the co‐location of resources, including CO_2_, hydrated cellular machinery, and light. Water and light are finite resources in many landscapes, leading to competition that is expected to favour traits that enhance the competitive ability of a given species under its native conditions (Givnish 1986a, 1986b; Smith et al. 2023). Uneven distributions of resources across landscapes ensure that traits that enhance competitive ability in one environment may not do so elsewhere (Givnish et al. 2004; Smith et al. 2023; Westerband et al. 2023). Traits of a given species should thus reflect adaptation to the resources available and competition for them in their native environments. Carbon gain is typically favoured in environments with greater moisture supply (Givnish 1986a; Huxman et al. 2004; Smith et al. 2023).

On upland sites, greater moisture supply can result from greater atmospheric input (annual precipitation, P) and/or lower atmospheric demand (annual pan evaporation, $E_{p}$), where $E_{p}$ integrates influences of temperature, humidity, wind and solar radiation (Rotstayn et al. 2006). The ratio $P/E_{p}$ thus quantifies the climatically determined ratio of moisture supply to demand (Smith et al. 2023). Greater $P/E_{p}$ better supports carbon gain as leaves with open stomata lose less water and/or lost water is more readily replaced from soil reserves. Both help maintain cellular hydration. Greater hydraulic conductance can support faster rates of carbon gain for any given soil type and climate. Adequate cellular hydration will reduce the need for symplastic solutes, thus resulting in less negative TLP (Kishor et al. 1995). Faster rates of carbon gain and growth in higher $P/E_{p}$ landscapes may, however, increase spatial crowding and competition for light, favouring more height growth, particularly in shade‐intolerant species (Givnish 1982, 1995; Tilman 1988). Taller plants typically display greater hydraulic conductivity (Petit et al. 2010; Poorter et al. 2010; Zach et al. 2010; Fan et al. 2012). Xylem with greater hydraulic conductivity is more porous and carries reduced carbon cost per volume, realized as lower tissue density and lower cost for height growth (Castro‐Díez et al. 1998).

Low $P/E_{p}$ environments should generally push trends in the opposite direction. Low $P/E_{p}$ is less favourable to carbon gain because cells near open stomata lose water more rapidly, because soils lack adequate reserves to replace lost water, and because plants allocate more heavily to unproductive root or water storage tissue. Benefits of accumulating symplastic solutes may outweigh their carbon cost under low $P/E_{p}$ conditions. High hydraulic conductivity provides less benefit when water is scarce. Lower hydraulic conductivity can result from less xylem area occupied by vessels and therefore more area occupied by fibres, increasing xylem density in association with increasing resistance to embolism formation (Jacobsen et al. 2005, 2007). Fibres themselves may further increase xylem density owing to their higher fractional area occupied by the cell wall (Pratt et al. 2007). In addition to the effects of hydraulic conductance, high xylem density may confer greater longevity through greater resistance to hydraulic dysfunction and mechanical damage (Martínez‐Vilalta et al. 2010). At the landscape scale, reduced carbon gain can alleviate crowding and thus aboveground competition and the necessity for height growth. Height can become a liability as it increases hydraulic transport distances and may expose canopies to more drying winds (Nagashima and Hikosaka 2011; Givnish et al. 2014). However, greater height can allow canopies to escape threats of fire (Grady and Hoffmann 2012), herbivores (Staver and Bond 2014), and competition from other plants.

Resources vary temporally as well as spatially. Trait plasticity provides the flexibility required for a genotype to maintain suitable trait combinations for survival and competition across geographic ranges and time scales. With $P/E_{p}$ variation, plasticity may be expected to broadly shift traits in the directions detailed above. However, plasticity has limitations (DeWitt et al. 1998; Givnish 2002). The extent of plastic change is itself mostly under genetic control, and not all organisms are capable of the same degree of plastic change. To be beneficial, plastic changes need to be timely and in the correct direction, requiring accurate environmental sensing and prediction (Karban and Adler 1996). This timeliness should be in response to the environment as well as with respect to the lifespan of a trait. For example, biochemical changes are likely more labile than structural changes. Costs of plastic changes could be small (e.g., osmolyte synthesis) or large (e.g., branch growth). Future trait changes may be limited by current traits, as woody perennial plants especially are shaped by past responses to available space and resources. Lastly, plastic trait changes may need to be coordinated for any or all to be effective. We thus expect trait plasticity to vary by trait and by species to suit trait lifespans and the environments to which each species has adapted.

Determining how traits differ between species and environments, and how traits shift with environment, is important for understanding the evolution of those traits and their importance for survival and competition. In this study, we examined traits in the genus *Eucalyptus* with respect to a steep gradient in $P/E_{p}$ across Victoria in SE Australia (Givnish et al. 2014) spanning environments from tall wet sclerophyll forests (home to the world's tallest angiosperm species, *Eucalyptus regnans*) to the semi‐arid mallee. *Eucalyptus* species are overwhelmingly evergreen and endemic to Australia, occupying all its ecoregions and exhibiting vast adaptation across species and environments. The two largest subgenera are *Eucalyptus* and *Symphyomyrtus*. Subgenus *Symphyomyrtus* underwent a rapid radiation beginning 2–3 MA ago, corresponding to a drying trend on the continent (Thornhill et al. 2019), and tends to be distributed on drier sites than subgenus *Eucalyptus* (Hughes et al. 1996).

Because *Eucalyptus* is a large and economically important genus with a broad climatic distribution, it is widely studied, and it is well recognized that traits within its taxa may correlate with the climate of origin, and these traits may shift plastically with the environment. Numerous studies have contributed to quantifying this trait variation through the use of common gardens (Warren et al. 2006; Turner et al. 2010; Bourne et al. 2017; Smith et al. 2023; Pan et al. 2025), wild populations (Pfautsch et al. 2016; Anderegg et al. 2021) or both (McLean et al. 2014; Britton et al. 2026). These studies point towards *Eucalyptus* species from drier and/or warmer climates having (among a range of differences): narrower xylem conduits that may or may not impact hydraulic conductivity (Pfautsch et al. 2016; Bourne et al. 2017; Pan et al. 2025); denser wood (Pfautsch et al. 2016; Britton et al. 2026); more negative TLP (Bourne et al. 2017); greater area‐specific leaf mass and nitrogen content (McLean et al. 2014; Britton et al. 2026). Typically, studies involving conspecifics at more than one location find that interspecific trait‐environment relationships differ with location. While informative, studies that rely on wild plants or that compare species planted at a single site do not control for within‐species variation, making it difficult to fully separate plastic variation from genetically fixed variation. Only by using the same genetic stock at multiple gardens can we begin to resolve this issue. However, studies incorporating more than one common garden are rare. Studies of genetically identical species grown at more than one site (e.g., McLean et al. 2014) can be informative but typically lack the range of species and sites required for robust statistical analysis.

To simultaneously examine effects of climate on plastic and genetically fixed trait variation in *Eucalyptus*, we established four common gardens across Victoria from $P/E_{p}=$ 0.16 to 1.25 ${\text{mm mm}}^{-1}$ (Smith et al. 2023; Table 1). We populated each common garden with 10 species, consisting of 5 from subgenus *Eucalyptus* and 5 from *Symphyomyrtus*. The native ranges of chosen species spanned our $P/E_{p}$ gradient (Table 2). Using this experimental design, we broadly ask how three traits that support and are related to carbon gain (hydraulic conductivity, xylem density, and TLP) relate to native $P/E_{p}$ of each species and how they change plastically with $P/E_{p}$ of sites (common gardens). At a finer scale, we explore how xylem anatomy (conduit diameter, conduit density and fibre wall fraction) varies with species $P/E_{p}$ and site $P/E_{p}$ and the extent to which xylem anatomy variation explains hydraulic conductivity and xylem density variation. Lastly, we ask how our three main traits correlate with each other independently of site and species $P/E_{p}$. We test six specific hypotheses:

**Table 1:** Common garden locations with elevation, soil, mean precipitation P, pan evaporation ($E_{p}$) and their ratio.

Site	Location	Elev (m)	Soil class	P (mm)	$E_{p}$ (mm)	$P/E_{p}$
Hattah	−34.74° N 142.25° E	42	Calcarosol	316	1983	0.16
Bealiba	−36.73° N 143.61° E	280	Sodosol	574	1475	0.39
Mt. Disappointment	−36.39° N 145.14° E	650	Dermosol	1256	1224	1.03
Toolangi	−36.49° N 145.57° E	688	Dermosol	1417	1132	1.25

*Note:* Listed in order of increasing $P/E_{p}$. Site names reflect the state forests in which they are located. Soil classes from the Bureau of Rural Sciences (2009).

**Table 2:** Study *Eucalyptus* species and climate data.

Species	Subgenus	P (mm)	$E_{p}$ (mm)	$P/E_{p}$
*Eucalyptus dumosa*	*Symphyomyrtus*	350	1930	0.19
*Eucalyptus arenacea*	*Eucalyptus*	546	1526	0.37
*Eucalyptus microcarpa*	*Symphyomyrtus*	594	1690	0.37
*Eucalyptus sideroxylon*	*Symphyomyrtus*	681	1693	0.42
*Eucalyptus macrorhyncha*	*Eucalyptus*	797	1453	0.58
*Eucalyptus viminalis*	*Symphyomyrtus*	855	1324	0.68
*Eucalyptus dives*	*Eucalyptus*	884	1315	0.69
*Eucalyptus obliqua*	*Eucalyptus*	954	1251	0.82
*Eucalyptus nitens*	*Symphyomyrtus*	1014	1219	0.85
*Eucalyptus regnans*	*Eucalyptus*	1043	1107	0.98

*Note:* Values are means. Listed in order of increasing $P/E_{p}$.

Within sites, species with higher $P/E_{p}$ should have higher hydraulic conductivity, lower wood density, less negative TLP, larger and fewer xylem conduits, and smaller fibre wall fraction.

Across sites, traits in H1 will plastically respond to increasing site $P/E_{p}$ in the same directions as in H1.

Species with higher $P/E_{p}$ should have greater plasticity of hydraulic conductivity, density, and xylem anatomy but lower plasticity of TLP.

Xylem anatomy will explain variation in hydraulic conductivity and xylem bulk density.

Given expected trait‐environment correlations of hydraulic conductivity, xylem density and TLP with $P/E_{p}$ (H1 and H2), we also expect these three traits to correlate with each other, which may explain residual variation in trait‐environment relationships.

Across all hypotheses, we had no *a priori* expectation that subgenus membership would explain residual variation. However, the tendency for subgenus *Symphyomyrtus* species to naturally occupy lower $P/E_{p}$ locations suggests that many traits may reflect this tendency.

## 2 Materials and Methods

### 2.1 Plant Material

Plant samples were collected from four common gardens in Victoria, Australia, spanning relative moisture supply ($P/E_{p}$) from 0.16 to 1.25 mm mm^−^^1^ (see Table 1). Each garden consisted of a fenced 0.25 ha plot established in 2016 within State Forests. All garden sites are upland (at least 4.8 m above the water table; https://datashare.maps.vic.gov.au/dataset ANZVI0803004920) surrounded by mature forests or woodland. Sites were planted with 10 species of *Eucalyptus* chosen from 2 subgenera (*Eucalyptus* and *Symphyomyrtus*) that each span the climate gradient (Smith et al. 2023; Table 2). Plants were raised from seed by Australian Native Farm Forestry (Cobram East, VIC) and transplanted to the sites in June 2018 at 6 months of age. Seedlings were planted in a 0.5 m grid through holes in fabric weed matting (Permethane, Campbelltown, NSW). At the two drier sites, we applied *ca*. 2 mm of irrigation at the time of planting. The driest site received this amount of water once more, 1 month after planting. The current study began 68 weeks after planting when site‐average heights were 43 cm (Hattah), 133 cm (Bealiba and Mt Disappointment) and 197 cm (Toolangi).

### 2.2 Site and Species Climate

We used data from the Atlas of Living Australia (ALA), Worldclim, and the Bureau of Meteorology (BOM) to determine long‐term climates of the common gardens and also the broad climate envelopes of the study species within their Australian ranges. For each species, we obtained occurrence data from ALA and then reduced sampling bias by rounding geographic coordinates to the nearest 0.25° and removing duplicates of each species‐location combination. We visualized these distributions and removed three outlier points averaging more than 5° from any conspecific. For each location, we extracted mean pan evaporation ($E_{p}$) from BOM (http://www.bom.gov.au/jsp/ncc/climate_averages/evaporation/index.jsp?period=an#maps) and mean annual precipitation (P) from Worldclim (Fick and Hijmans 2017).

### 2.3 Hydraulic Conductivity

We collected samples from October to December 2019. At that time, *Eucalyptus dives*, *Eucalyptus obliqua* and *E. regnans* at Hattah either had failed or were so reduced in number that collection would compromise other measurements. For each remaining species at each site, we selected typically five plants at random, cut one branch in the air, immediately placed it in a moistened plastic bag, and kept it away from heat until it could be refrigerated later that day. Selected branches were healthy non‐leaders with basal diameters between 2 and 5.2 mm and with minimal side branching.

Within 3 days of collection, from each branch, we excised an 11 cm segment for hydraulic conductivity (K) measurement and removed any leaves or side branches. Stems were likely shorter than the longest vessel, but K is not sensitive to open vessels over a wide length range (Melcher et al. 2012). We cut stem ends underwater with a razor blade, and then vacuum infiltrated them in distilled water overnight to maximize conductivity (Espino and Schenk 2011). After infiltration, we cut the ends again and then tightly wrapped each stem with Parafilm where leaves or stems had been removed. For each stem, we measured at least three steady‐state flow rates (Q; $g\;s^{-1}$) at two or more pressure potentials (*ψ*) between *ca*. 0.4 and 4.9 kPa. Multiple pressures were used to be able to account for passive uptake (Torres‐Ruiz et al. 2012). We measured flow rates using a KCl solution flowing through a flow metre (Sack et al. 2011). We used a CR6 datalogger (Campbell Scientific, Logan, UT) to log pressures (PX26‐005GV, Omega Engineering, Norwalk, CT) and solution temperature (Type‐T thermocouple) every 1 s. We averaged readings for 10 s after reaching stability.

We calculated each K (g mm s^−^^1^ MPa^−^^1^) as the slope of a linear regression fit to Q versus ψ/L, where L is stem length (mm). We standardized K to 20°C and then divided this by xylem area ($A_{\mathrm{xyl}}$; mm^2^) to obtain xylem specific conductivity, $K_{x}$ ($g\;s^{-1}\;{\text{MPa}}^{-1}\;{\text{mm}}^{-1}$). For $A_{\mathrm{xyl}}$, we measured xylem diameter with callipers, calculated the area of the equivalent circle, and then subtracted pith area measured from microscopy (see Section 2.6).

### 2.4 Xylem + Pith Density

For each stem used for $K_{x}$, we excised a *ca*. 4 cm length segment, measured diameter, removed tissues superficial to xylem and then measured volume by displacement on a 0.001 g precision balance and recorded mass after drying at 70°C to determine density. Because the samples contain xylem and pith, we symbolically call this $\rho_{x,p}$. We collected additional density samples from the same plantings at two later time frames. For both timeframes, we selected seven plants per species, spanning their height range at the site. For each plant, we excised a section of the trunk, measured the diameter, and then determined the density as above. For the earlier collection, we also obtained density measurements from branches, chosen to obtain a wide variety of stem diameters. In total, we measured 893 samples with typically n=26 per site*species combination, and all possible combinations represented except *E. dives* and *regnans* at Hattah, where none survived.

### 2.5 TLP

Using material separate from $K_{x}$ measurements, one healthy shoot was collected from up to seven plants per species per site in March 2021. By this time, failures had occurred for *Eucalyptus arenacea*, *dives*, *macrorhyncha*, *nitens*, *obliqua* and *regnans* at Hattah and *E. dives* at Bealiba. Shoots were immediately bagged, sprayed with water, and soon stored in a portable refrigerator until measurement within 4 days. For each shoot, we took a 16 mm punch from a lamina, avoiding major veins, and placed this in distilled water for 2 to 3 h. A preliminary test showed that disk mass increased to a stable value within this time. We measured osmotic potential similar to Bartlett et al. (2012). In short, we removed surface water from each disk, subsampled a 6‐mm disk to exclude free water at disk margins, froze aluminium foil‐wrapped disks in liquid nitrogen for at least 2 min, placed each thawed disk in the cap of a calibrated cage psychrometer chamber (75‐3VC, JRD Merrill, Logan UT; Brown and Bartos 1982), repeatedly pricked its surface, and sealed this cap to the psychrometer. We submerged bagged chambers in a room‐temperature water bath and measured water potential every 20 min with a CR6 datalogger. After 1 h equilibration, we averaged the four readings from 1 to 2 h.

### 2.6 Xylem Anatomy

For all stems used for $K_{x}$, we rehydrated dried samples overnight before cross‐sectioning, staining with toluidine blue and photographing for measurement. We chose measurements to ask how anatomy varies across sites and taxa, and how anatomy explains $K_{x}$ and $\rho_{x,p}$. From images taken with a 4x objective, we measured pith area ($A_{\mathrm{pith}}$). From images taken with a 10x objective, we measured the lumen areas of all conduits ($A_{\mathrm{lum}}$) in the largest available area of interest (AOI), bound by cambium and rays going as deep towards pith as each image allowed. We calculated: hydraulically weighted average conduit diameter [$D_{\mathrm{HW}}={\left(\frac{1}{n}\sum A_{\mathrm{lum}}^{2}\right)}^{0.25}$, where n is the number of conduits]; conduit density (CD=n/AOI); and non‐conduit fraction (i.e., xylem area fraction occupied by fibres, rays and parenchyma: $1-\frac{\sum A_{\mathrm{lum}}}{\text{AOI}}$) as a proxy for xylem area occupied by fibres ($A_{\mathrm{fib}}/A_{\mathrm{xyl}}$). From images taken with a 40x objective, we measured 10 fibres at locations where random points fell. We calculated the fibre wall fraction from the ratio of wall area to cell area ($A_{\mathrm{wall}}/A_{\mathrm{fib}}$). We averaged this fraction across each image and then averaged across at least two images per stem.

Since density samples contained xylem and pith, measured density depends on respective densities (ρ) of xylem and pith and their relative areas in cross‐section:

(1) $$\rho_{x,p}=\rho_{\mathrm{xyl}}\frac{A_{\mathrm{xyl}}}{A_{x,p}}+\rho_{\mathrm{pith}}\frac{A_{\mathrm{pith}}}{A_{x,p}}.$$

Xylem area and xylem density can be further separated into contributions from cell walls and lumens. If $A_{\mathrm{xyl}}=A_{\mathrm{wall}}+A_{\mathrm{lum}}$, then,

(2) $$\rho_{\mathrm{xyl}}=\rho_{\mathrm{wall}}\frac{A_{\mathrm{wall}}}{A_{\mathrm{xyl}}}+\rho_{\mathrm{lum}}\frac{A_{\mathrm{lum}}}{A_{\mathrm{xyl}}}=\rho_{\mathrm{wall}}\frac{A_{\mathrm{wall}}}{A_{\mathrm{xyl}}},$$

where the second equality exists when lumen density is 0. Equations (1) and (2) show:

(3) $$\rho_{x,p}=\rho_{\mathrm{wall}}\frac{A_{\mathrm{wall}}}{A_{x,p}}+\rho_{\mathrm{pith}}\frac{A_{\mathrm{pith}}}{A_{x,p}}.$$

$A_{\mathrm{wall}}/A_{x,p}$ is not readily measurable directly but can be estimated as the product of the area fractions of xylem in sample ($A_{\mathrm{xyl}}/A_{x,p}$), fibres in xylem ($A_{\mathrm{fib}}/A_{\mathrm{xyl}}$), and cell wall in fibres ($A_{\mathrm{wall}}/A_{\mathrm{fib}}$):

(4) $$\frac{A_{\mathrm{wall}}}{A_{x,p}}=\frac{A_{\mathrm{xyl}}}{A_{x,p}}\frac{A_{\mathrm{fib}}}{A_{\mathrm{xyl}}}\frac{A_{\mathrm{wall}}}{A_{\mathrm{fib}}}.$$

According to Equation (3), $\rho_{x,p}$ variation should be driven by individual densities and fractions of sample areas occupied by fibre walls and by pith. Equation (4) further suggests that the area occupied by fibre walls could be driven by one or more component fractions.

### 2.7 Size Corrections

After K values were divided by $A_{\mathrm{xyl}}$ (making $K_{x}$), some species had significant correlations between $K_{x}$ and $A_{\mathrm{xyl}}$ (Supporting Information S1: Figure S1). This is expected as $K_{x}$ should increase faster than $A_{\mathrm{xyl}}$ due to packing and taper functions of the xylem (Savage et al. 2010) coupled with unavoidable variation in $A_{\mathrm{xyl}}$ across sites, species and individuals. Failure to address this issue can confound size differences with $K_{x}$ differences. As a solution, we fit each species with the model ${\log(K}_{x})\sim \log(A_{\mathrm{xyl}})+\mathrm{site}$. Using each species' slope, and each individual's observed $K_{x}$ and $A_{\mathrm{xyl}}$, we calculated $K_{x}$ at the study's median interspecific $A_{\mathrm{xyl}}$ (Supporting Information S1: Figure S1). Corrected values are denoted with a dot: ${\dot{K}}_{x}$.

Taper and packing functions also mean that conduit diameter and density are prone to confounding size effects. Therefore, we corrected $D_{\mathrm{HW}}$ and CD with the same method above, except using stem diameter instead of xylem area (Supporting Information S1: Figures S2 and S3). Corrected values are denoted as ${\dot{D}}_{HW}$ and $\dot{CD}$. $A_{\mathrm{wall}}/A_{\mathrm{fib}}$ did not need correction.

We standardized $\rho_{x,p}$ by $D_{\mathrm{stem}}$ for each site*species combination by first binning diameters every $2^{i}$ mm (bin boundaries at 2, 4, 8 … 128 mm) and then averaging density and diameter within bins to more equally weight many small diameter samples and few large diameter samples. Using these mean, binned values, we fit the regression $\rho_{x,p}\sim D_{\mathrm{stem}}+\mathrm{site}$ to each species. Regressions returned species‐specific slopes, which we used to scale individual densities to the global median diameter. Corrected values are denoted as ${\dot{\rho }}_{x,p}$.

### 2.8 Analyses

We conducted all analyses in R 4.3.0 (R Core Team 2023). Functions and general approaches mentioned with one hypothesis apply to subsequent hypotheses.

To test H1 (traits vs. species $P/E_{p}$), we averaged measurements of ${\dot{K}}_{x}$, ${\dot{\rho }}_{x,p}$, TLP and xylem anatomy within each site*species combination. We regressed these means against species $P/E_{p}$ with site and subgenus as fixed effects using *stats::lm* (R *package::function*). We considered additive and interactive models, with log transformation and without. When choosing between models, we made comparisons based on adjusted $R^{2}$ values, parameter significance (*stats::ANOVA*), AIC (*stats::AIC*) and parsimony. All reported *p*‐values come from two‐tailed tests. We assessed model slopes as $\frac{d\text{trait}}{\mathrm{dP}/E_{p,\mathrm{species}}}$, obtained from model derivatives. In models with log‐transformed species $P/E_{p}$, the slope was a function of species $P/E_{p}$. To report a mean slope for these models, we predicted trait values at the extremes of species $P/E_{p}$ and calculated the slope of a straight line between these values (Supporting Information S1: Figure S4). Slopes have units of trait units per mm mm^−1^, which we abbreviate to trait units. We made Tukey‐adjusted pairwise comparisons of intercept and slope coefficients (*emmeans::emmeans*, *emtrends*; Lenth 2023). After choosing the best fixed‐effects model, we asked if species and phylogeny were significant additions as random effects (*phyr::pglmm*; Ives et al. 2020), using a phylogeny from Thornhill et al. (2019) and handled with *ape* (Paradis and Schliep 2019).

We tested H2 (trait plasticity) with two models for each trait. First, we made ‘full’ models by regressing trait means against site $P/E_{p}$ and species $P/E_{p}$ simultaneously, considering the interaction between them and subgenus as a fixed effect. For each best model, we tested random effects for site, species and phylogeny. Second, we made ‘individual’ models by regressing trait means against site $P/E_{p}$, allowing species‐specific slopes and intercepts.

We tested H3 (differential trait plasticity) using models from H2. We assessed plasticity as $\frac{d\text{trait}}{\mathrm{dP}/E_{p,\mathrm{site}}}$, which we obtained from partial derivatives of H2 models for each species. In models with log‐transformed site $P/E_{p}$, we found the mean slope (plasticity) as described for H1. We considered models to support differential plasticity if plasticity trended upward or downward with species $P/E_{p}$.

We tested H4 (anatomy explaining $K_{x}$ and $\rho_{x,p}$) using individual measurements from samples from which we measured $K_{x}$, $\rho_{x,p}$ and xylem anatomy. We regressed $K_{x}$ against theoretical conductivity ($K_{x,\mathrm{theo}}$) calculated using the Hagen–Poiseuille equation (Tyree and Zimmermann 2002). We tried site, species and subgenus as factors. We then tried $D_{\mathrm{HW}}$ and CD as predictors in place of $K_{x,\mathrm{theo}}$. These predictions of $K_{x}$ used log‐transformed variables only. We regressed $\rho_{x,p}$ against $A_{wall}⁄A_{x,p}$ plus $A_{pith}⁄A_{x,p}$ without intercept (Equation 3). Excluding an intercept allows us to interpret regression coefficients as estimates of $\rho_{\mathrm{wall}}$ and $\rho_{\mathrm{pith}}$. In no‐intercept regressions, we calculated $R^{2}$ as 1−RSS/TSS (i.e., residual over total sum of squares) because *stats::lm* returns abnormally high $R^{2}$. We tested if site, species, and subgenus interact with $A_{wall}⁄A_{x,p}$, effectively testing if $\rho_{\mathrm{wall}}$ differs across these factors. We then repeated regressions, replacing $A_{wall}⁄A_{x,p}$ with its three components (Equation 4).

We tested H5 (trait covariation) by regressing means of ${\dot{K}}_{x}$, ${\dot{\rho }}_{x,p}$ and TLP against each other, with and without log transformation, while trying site, species and subgenus as factors. To determine if these traits could explain variation left unexplained in each other's site $P/E_{p}$ and species $P/E_{p}$ model (i.e., H2), we first re‐ran each model using a restricted dataset where ${\dot{K}}_{x}$ and ${\dot{\rho }}_{x,p}$ have fewer points to match the TLP data. For these models, we tried adding a trait and tried replacing the site $P/E_{p}$ or species $P/E_{p}$ terms with that trait.

All of the above analyses considered the subgenus as a factor to test H6.

## 3 Results

### 3.1 Genetically Fixed Trait Patterns

#### 3.1.1 Conductivity

Within all four common gardens, hydraulic conductivity (${\dot{K}}_{x}$) increased with species $P/E_{p}$ (both log‐transformed, *p* < 0.001; Figure 1A–D, Supporting Information S2: Table S1). Across sites, slopes were conserved (*p* = 0.54), but intercepts differed significantly (*p* < 0.001), increasing in order of site $P/E_{p}$. That is, ${\dot{K}}_{x}$ generally increased at a given species $P/E_{p}$ as site $P/E_{p}$ increased (Figure 1A–D, Supporting Information S2: Table S1). Overall, $R^{2}=0.75$. Subgenus had no observed effect on slope (*p* = 0.32) or intercept (*p* = 0.19). Supporting Information S2: Table S2 shows all model contrasts. Random effects were not significant for phylogeny (*p* = 1) or species (*p* = 0.75). Phylogeny was not a significant addition for any trait (*p* > 0.97), so it will not be mentioned further.

**Figure 1:**
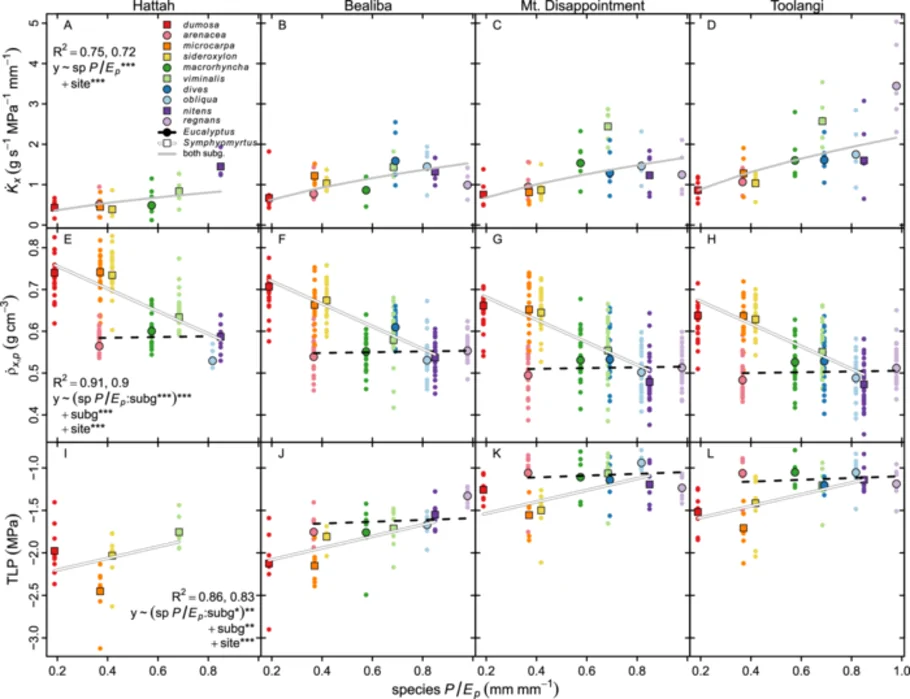
Hydraulic conductivity, xylem + pith density and turgor loss point all correlated with species $P/E_{p}$ (precipitation per pan evaporation) across sites (columns). For all panels, large points are the means to which regressions were fit. Small points are individual measurements. Continuous lines indicate slopes with *p* < 0.05 and dashed lines indicate otherwise. Text in the first column lists model details for the row, including multiple and adjusted $R^{2}$ values, model parameters and the significance of their removal. (A–D) Standardized specific hydraulic conductivity (${\dot{K}}_{x}$; see Section 2) increased with species $P/E_{p}$ at all sites, using log‐transformed variables. The rate of increase did not differ between sites or subgenera, but intercepts differed between sites, tending to increase from the driest site to the wettest (left to right). (E–H) Standardized xylem + pith density (${\dot{\rho }}_{x,p}$; see Section 2) declined with species $P/E_{p}$ but only in subgenus *Symphyomyrtus*. Intercepts differed by subgenus and by site, decreasing in order from the driest site to the wettest. (I–L) Turgor loss point increased with species $P/E_{p}$ in subgenus *Symphyomyrtus* but not in subgenus *Eucalyptus*. Slopes did not differ between sites, but intercepts did, tending to increase from the driest site to the wettest.

#### 3.1.2 Xylem + Pith Density

Mean ${\dot{\rho }}_{x,p}$ ranged from 0.47 ${\text{g cm}}^{-3}$ (*E. nitens* at Toolangi) to 0.74 ${\text{g cm}}^{-3}$ (*E. microcarpa* at Hattah). Within sites, ${\dot{\rho }}_{x,p}$ declined sharply with species $P/E_{p}$ within subgenus *Symphyomyrtus* but not in *Eucalyptus* (Figure 1E–H; Supporting Information S2: Table S1). Intercepts differed between sites (*p* < 0.001), decreasing as site $P/E_{p}$ increased. Overall, $R^{2}=0.91$.

Species as a random effect was a significant addition (*p* < 0.001). Its addition tended to increase predicted density for moderate $P/E_{p}$ (*E. microcarpa*, *sideroxylon*, *macrorhyncha*, *viminalis*, *dives*) and decrease predictions at extremes of $P/E_{p}$.

#### 3.1.3 TLP

TLP increased significantly with species $P/E_{p}$ in subgenus *Symphyomyrtus* but not *Eucalyptus* (Figure 1I–L; Supporting Information S2: Table S1). Intercepts differed between sites (*p* < 0.001) and increased roughly in order of site $P/E_{p}$. Overall, $R^{2}=0.86$. Species as a random effect was significant (*p* = 0.04). Its addition primarily predicted less negative values for *E. dumosa* and more negative values for *microcarpa*.

#### 3.1.4 Xylem Anatomy

Within sites, conduit diameter increased with species $P/E_{p}$ (Figure 2A–D) while conduit density declined with log‐transformed species $P/E_{p}$ (Figure 2E–H, Supporting Information S2: Table S3). For both traits, slopes differed by site (*p* < 0.001). Conduit diameter slopes also differed by subgenus (*p* < 0.001). Conduit density intercepts differed by subgenus (*p* < 0.001). Overall, $R^{2}=0.79$ (conduit diameter) and 0.89 (conduit density). Together, models showed wider and fewer conduits in subgenus *Symphyomyrtus*. In subgenus *Eucalyptus*, conduit diameter slope (per species $P/E_{p}$) was between 5.6 μm (Hattah) and 20.3 μm (Toolangi). In subgenus *Symphyomyrtus*, these slopes were 8.3 μm steeper. Conduit density was offset 17.49 mm^−2^ lower in subgenus *Symphyomyrtus*. Conduit density's average slope was shallowest at Hattah (−44.4 mm^−2^) and steepest at Toolangi (−147.8 mm^−2^). Pairwise comparisons of conduit density slope grouped the first‐ and third‐driest sites separately from the second‐ and fourth‐driest sites.

**Figure 2:**
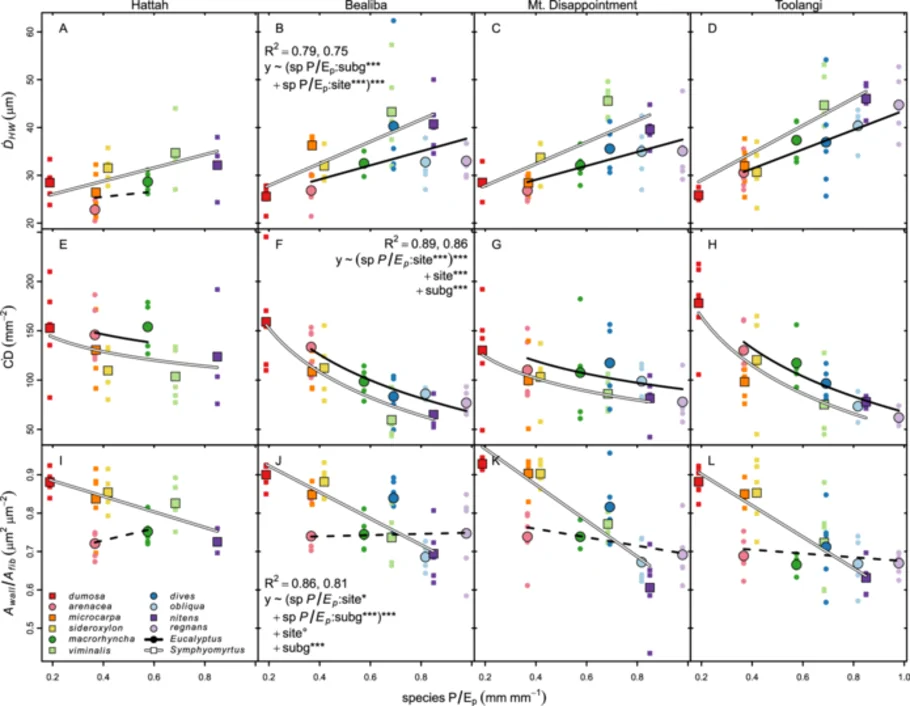
Xylem anatomical traits all correlated with species $P/E_{p}$ (precipitation per pan evaporation) across sites (columns). For all panels, large points are the means to which regressions were fit. Small points are individual measurements. Solid lines indicate slopes with *p* < 0.05 and dashed lines indicate otherwise. Text in the second column lists model details for the row, including multiple and adjusted $R^{2}$ values, model parameters and the significance of their removal. (A–D) Hydraulically weighted mean conduit diameter increased with species $P/E_{p}$. Slopes differed between sites and between subgenera, with steeper slopes at sites with higher $P/E_{p}$ (left to right) and among subgenus *Symphyomyrtus*. (E–H) Conduit density somewhat mirrored the patterns of conduit diameter. Conduit density decreased with log species $P/E_{p}$. Slopes and intercepts differed between sites, and intercepts differed further by subgenus, showing *Symphyomyrtus* had consistently lower conduit density. While sites differed, a clear trend across sites did not emerge. (I–L) Fibre wall fraction declined with species $P/E_{p}$ in subgenus *Symphyomyrtus*. Slopes also differed by site, being shallowest at Hattah and steepest at Mt. Disappointment. [Color figure can be viewed at wileyonlinelibrary.com]

$A_{\mathrm{wall}}/A_{\mathrm{fib}}$ showed a pattern similar to that for ${\dot{\rho }}_{x,p}$, decreasing with species $P/E_{p}$ in subgenus *Symphyomyrtus* (*p* < 0.001 across sites) but lacking any significant trend in subgenus *Eucalyptus* (*p* = 0.95 across sites; Figure 2I–L; Supporting Information S2: Table S3). Slopes also differed by site (*p* = 0.043). Overall, $R^{2}=0.86$. In *Symphyomyrtus*, slopes became steeper from Hattah (−0.20) to Mt. Disappointment (−0.47). Negative slopes were attributable to a shrinking numerator (less wall area) and growing denominator (larger cells) with increasing site $P/E_{p}$. $A_{\mathrm{wall}}$ versus species $P/E_{p}$ slope was −6.9 μm^2^ and intercepts differed by site (*p* < 0.001), predicting that $A_{\mathrm{wall}}$ increased by 14.2 μm^2^ from its minimum at Hattah to its maximum at Mt. Disappointment. Meanwhile, $A_{\mathrm{fib}}$ versus species $P/E_{p}$ slope was site‐dependent (*p* < 0.001) between 7.7 μm^2^ (Hattah) and 49.8 (Mt. Disappointment). Together, these trends imply that fibre wall thickness decreased with species $P/E_{p}$.

Species as a random effect was a significant addition for $A_{\mathrm{wall}}/A_{\mathrm{fib}}$ (*p* < 0.001), but not for conduit density (*p* = 0.29) and was weak for conduit diameter (*p* = 0.09). Like the models for ${\dot{\rho }}_{x,p}$, addition of species as a random effect tended to increase predictions for species with mid $P/E_{p}$ for $A_{\mathrm{wall}}/A_{\mathrm{fib}}$ (*E. sideroxylon, viminalis, dives*) and conduit diameter (*E. macrorhyncha, viminalis, dives*).

### 3.2 Trait Plasticity

#### 3.2.1 Conductive Plasticity

${\dot{K}}_{x}$ increased plastically with site $P/E_{p}$ simultaneously with species $P/E_{p}$ (Figure 3A, Supporting Information S2: Table S1; $R^{2}=0.70$; all data log‐transformed). This model was not improved by adding random effects of species (*p* = 1) or site (*p* = 0.47). Log‐transformation of all variables resulted in ${\dot{K}}_{x}$ approximating a power function of site and species $P/E_{p}$:

(5) $${\dot{K}}_{x}=c{\left(P⁄E_{p,site}\right)}^{a}{\left(P⁄E_{p,species}\right)}^{b},$$

**Figure 3:**
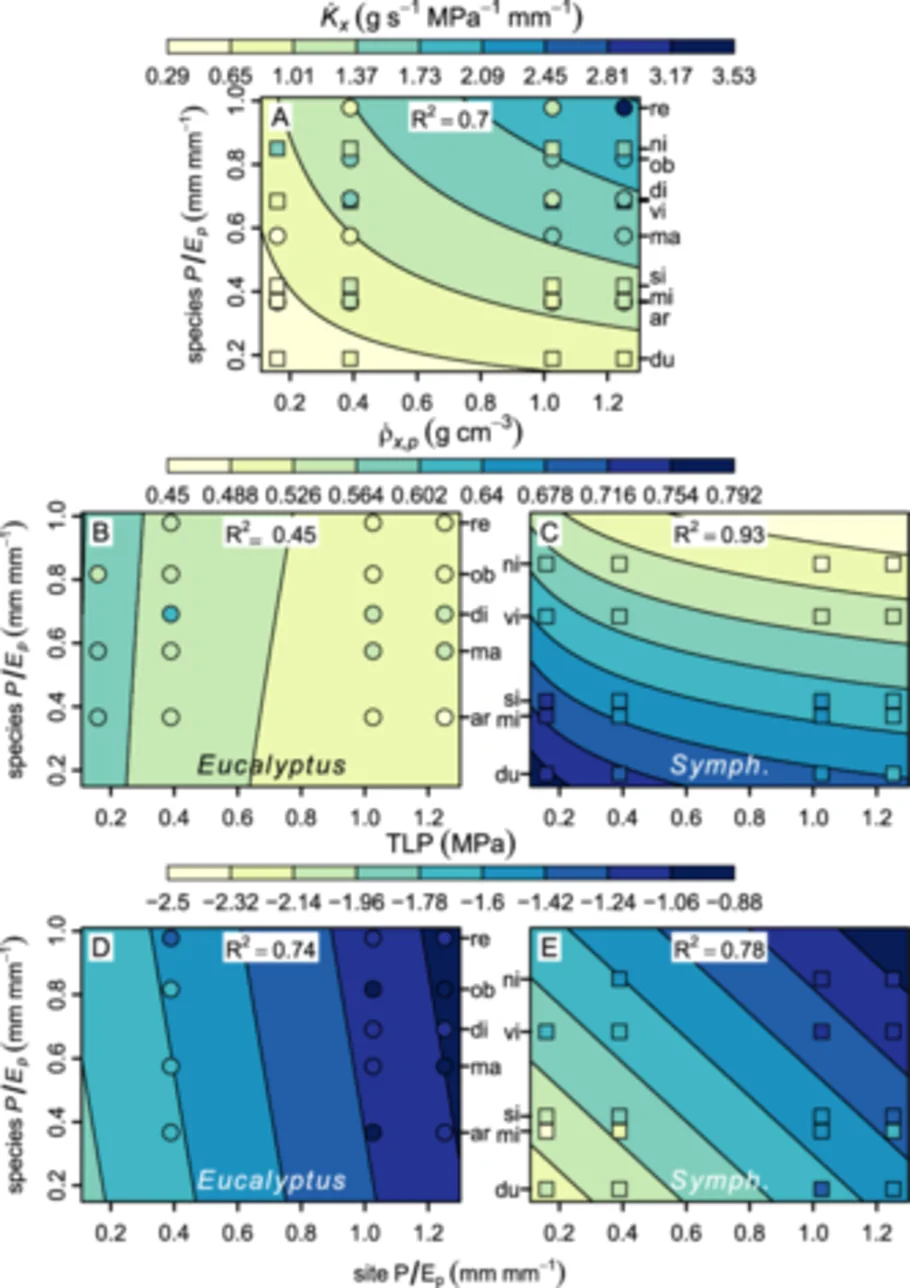
Hydraulic conductivity, xylem + pith density and turgor loss point changed plastically with site $P/E_{p}$ (precipitation per pan evaporation) simultaneously with species $P/E_{p}$. For all panels, points are observed means to which models were fit to create contours. Models are shown in two panels (left and right) when predictions differed by subgenus. Species names are indicated by their first two letters on the *y*‐axis. (A) Standardized specific hydraulic conductivity (${\dot{K}}_{x}$; see Section 2) increased simultaneously with site $P/E_{p}$ and species $P/E_{p}$ (all log‐transformed). Subgenera did not differ, and climate variables did not interact, but the log‐transformation of all variables meant that plasticity was a function of species $P/E_{p}$. (B and C) Standardized xylem + pith density (${\dot{\rho }}_{x,p}$; see Section 2) decreased with species $P/E_{p}$ differently between subgenera, similar to Figure 1E–H, but in both subgenera, it plastically declined at the same rate with site $P/E_{p}$. In subgenus *Symphyomyrtus*, plasticity was shallower than the effects of species $P/E_{p}$. Climate variables did not interact. Thus, plasticity did not differ between species. (D and E) Turgor loss point increased (became less negative) with species $P/E_{p}$ differently between subgenera, similar to Figure 1I–L, but in both subgenera, it plastically increased with site $P/E_{p}$ at the same rate. Climate variables did not interact. Thus, plasticity did not differ between species. [Color figure can be viewed at wileyonlinelibrary.com]

where a, b and c are fitting coefficients. Plasticity $\left(\frac{d{\dot{K}}_{x}}{dP⁄E_{p,site}}\right)$ is then a function of $P/E_{p}$ of site and species:

(6) $$\frac{d{\dot{K}}_{x}}{dP⁄E_{p,site}}=ca{\left(P⁄E_{p,site}\right)}^{a-1}{\left(P⁄E_{p,species}\right)}^{b}.$$

Thereby, ${\dot{K}}_{x}$ exhibited differential plasticity between species. Exponent b was positive, meaning that at all sites, plasticity increased with species $P/E_{p}$, in support of hypothesis 3 (Figure 5A). Exponent a was positive but less than 1, meaning that for each species, plasticity declined with site $P/E_{p}$. Individually, 6 of 10 species had significant, positive relationships between ${\dot{K}}_{x}$ and site $P/E_{p}$ (both log‐transformed; Supporting Information S2: Table S1). Significant exponents (akin to a in Equations 5 and 6) were also positive but less than 1. Mean ${\dot{K}}_{x}$ plasticity increased with species $P/E_{p}$, similarly to predictions by Equation (6) (Figure 5A).

#### 3.2.2 Xylem + Pith Density Plasticity

${\dot{\rho }}_{x,p}$ decreased plastically with log‐transformed site $P/E_{p}$ simultaneously with subgenus‐specific trends with species $P/E_{p}$, but these did not interact, and plasticity did not differ between subgenera ($R^{2}=0.91$; Figure 3B,C; Supporting Information S2: Table S1). Therefore, differential plasticity across species was not observed. Log‐transformation of site $P/E_{p}$ meant plasticity decreased inversely from low to high site $P/E_{p}$ (i.e., $\frac{d}{\mathrm{dx}}a\log(x)=a/x$; Supporting Information S1: Figure S4). Mean plasticity from Hattah to Toolangi was −0.076 ${\text{g cm}}^{-3}$, which is between slopes with species $P/E_{p}$ for subgenus *Symphyomyrtus* (−0.27) and *Eucalyptus* (0.009).

Species was a significant addition as a random effect (*p* < 0.001), altering predictions in the same pattern as it did for ${\dot{\rho }}_{x,p}$ versus species $P/E_{p}$. Site was not a significant random effect (*p* = 1). Individually, all 10 species exhibited plasticity, having significant negative relationships with log site $P/E_{p}$ (*p* < 0.01; total $R^{2}=0.99$; Supporting Information S2: Table S1). Species‐specific plasticities differed (*p* = 0.02), due to *E. dives* and *nitens* having steeper slopes than *obliqua*, but showed no trend with species $P/E_{p}$ (Figure 5B).

#### 3.2.3 TLP Plasticity

TLP increased plastically with site $P/E_{p}$ simultaneously with subgenus‐specific trends with species $P/E_{p}$, but these did not interact, and plastic responses did not differ by subgenus ($R^{2}=0.82$; Figure 3D,E, Supporting Information S2: Table S1). Thus, differential plasticity by subgenus or species was not supported. Plasticity slope was 0.63 MPa, similar to the slope with species $P/E_{p}$ in subgenus *Symphyomyrtus* (0.69) but greater than in subgenus *Eucalyptus* (0.11). We found no significant random effects of species (*p* = 0.27) or site (*p* = 0.19). Individually, 7 of 10 species had a significant, positive relationship with site $P/E_{p}$, but slopes did not differ (*p* = 0.60). No substantial trend was found across individual measures of plasticity (Figure 5C, Supporting Information S2: Table S1).

#### 3.2.4 Anatomical Plasticity

Conduit diameter increased plastically with log‐transformed site $P/E_{p}$ simultaneously with species $P/E_{p}$. These variables interacted weakly (*p* = 0.053), indicating differential plasticity. Intercepts differed by subgenus (*p* < 0.001), giving *Symphyomyrtus* 4.6 μm larger diameters. Overall, $R^{2}=0.71$ (Figure 4A,B; Supporting Information S2: Table S3). Plasticity was lowest in *E. dumosa*, ranging from 0.1 μm at Toolangi to 0.8 μm at Hattah, and highest in *regnans*, ranging from 4.3 μm (Toolangi) to 33.7 μm (Hattah). These ranges spanned the slope with species $P/E_{p}$ (23.3 μm). Adding random effects made no significant improvements (species: *p* = 0.18; site: *p* = 0.57). Individually, only 3 of 10 species had a significant relationship with log‐transformed site $P/E_{p}$ (*p* < 0.05). Slopes did not differ (*p* = 0.22), but individual plasticities increased with species $P/E_{p}$ (Figure 5D).

**Figure 4:**
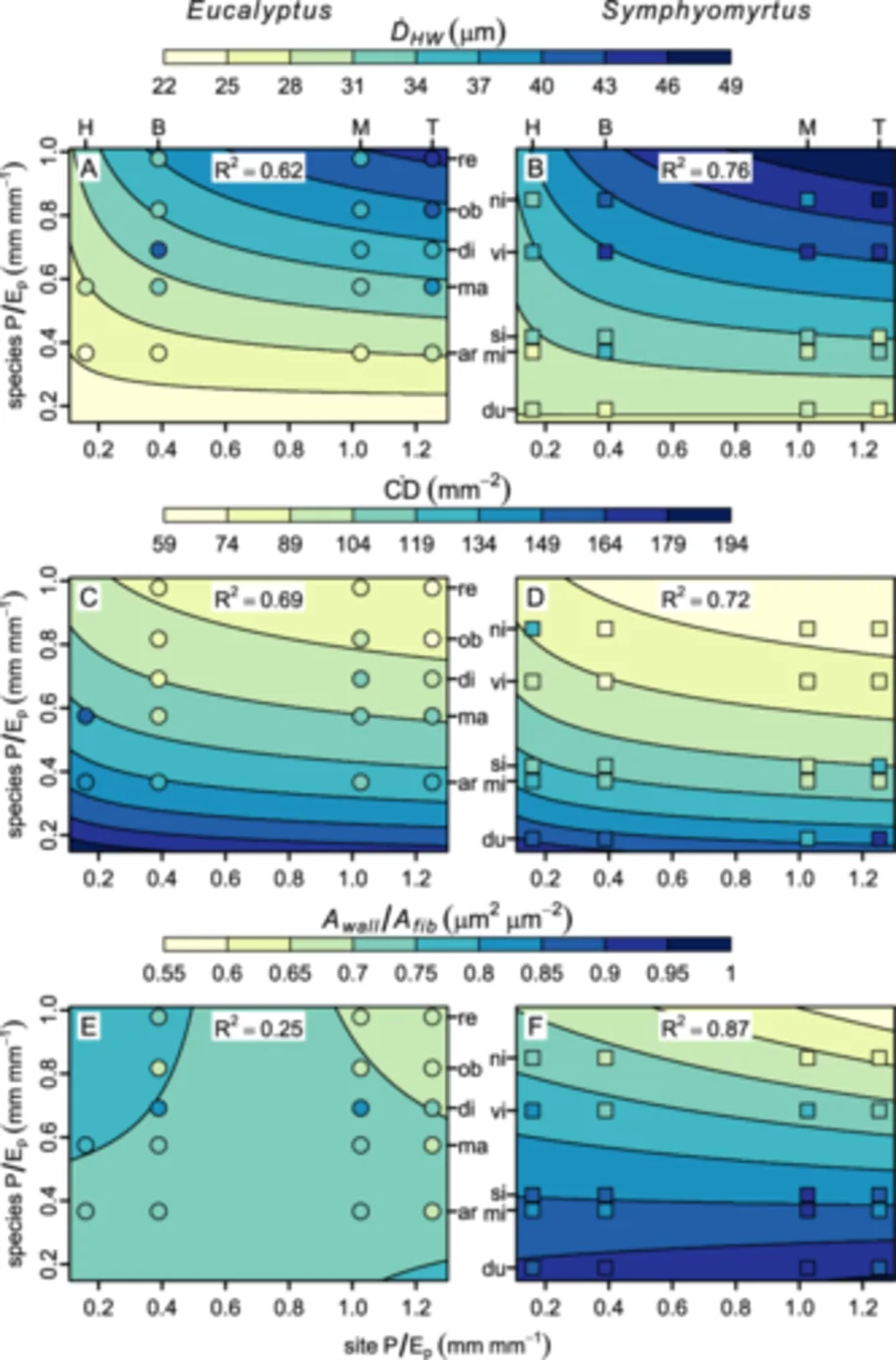
Xylem anatomy changed plastically with site $P/E_{p}$ (precipitation per pan evaporation) simultaneously with species $P/E_{p}$. For all panels, points are observed means to which models were fit to create contours. Models are shown in two panels (left and right) when predictions differed by subgenus. Species names are indicated by their first two letters on the *y*‐axis. (A and B) Standardized hydraulically weighted conduit diameter increased plastically with log site $P/E_{p}$. Subgenera did not show different plasticity, but they were offset such that *Symphyomyrtus* had wider conduits. Species $P/E_{p}$ and log site $P/E_{p}$ interacted, suggesting plasticity increased with species $P/E_{p}$. (C and D) Conduit density decreased with log site $P/E_{p}$. As suggested by Figure 2E–H, this trend was shallow compared to the effects of species $P/E_{p}$. Plasticity did not differ by species $P/E_{p}$ or by subgenus, but subgenera were offset such that *Symphyomyrtus* had lower conduit density. (E and F) Fibre wall fraction decreased with site $P/E_{p}$ on top of subgenus‐specific trends with species $P/E_{p}$. Plasticity did not differ by subgenus, but site $P/E_{p}$ and species $P/E_{p}$ interacted, suggesting plasticity was more pronounced as species $P/E_{p}$ increased. [Color figure can be viewed at wileyonlinelibrary.com]

**Figure 5:**
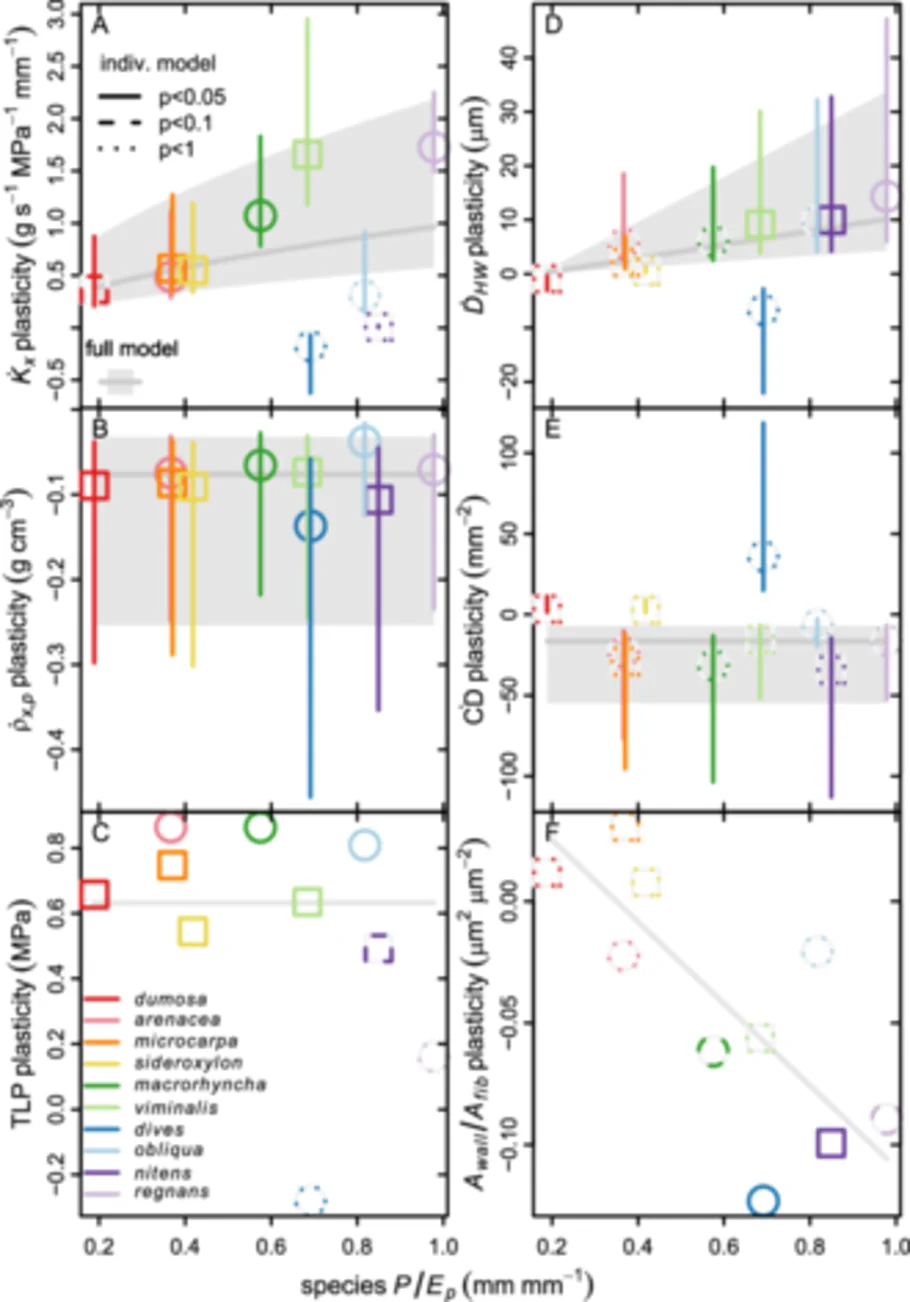
Plasticity $\left(\frac{d\text{trait}}{\mathrm{dP}/E_{p,\mathrm{site}}}\right)$ varied with species $P/E_{p}$ (precipitation per pan evaporation) in three of six traits. For all panels, grey lines are the mean predictions from models in Figures 3 and 4. Coloured open symbols are means from regression for each species. Symbol outlines are solid, dashed or dotted depending on significance (see legend). Log transformation of site $P/E_{p}$ for four traits meant that plasticity was site‐dependent, as represented by vertical span around symbols and grey lines. Plasticity units are technically as shown per mm mm^−1^. (A) ${\dot{K}}_{x}$ plasticity was positive and significant (*p* < 0.05) in six species (symbols). These values increased with species $P/E_{p}$ similarly to that predicted by the model in Figure 3A. (B) ${\dot{\rho }}_{x,p}$ plasticity was negative and significant in all species (*p* < 0.006; symbols). Species differed significantly (*p* = 0.02) but showed no trend with species $P/E_{p}$, consistent with predictions from the model in Figure 3B,C. (C) TLP plasticity was significant (*p* < 0.05) in seven species. Values were generally positive but lacked an apparent trend, consistent with the model shown in Figure 3D,E. (D) ${\dot{D}}_{HW}$ plasticity was significant in only three species. Values were generally positive and tended to increase from near zero as species $P/E_{p}$ increased, similar to the model shown in Figure 4A,B. (E) $\dot{\mathrm{CD}}$ plasticity was not significant for any species. Values were mostly negative and showed no notable trend with species $P/E_{p}$, consistent with the model shown in Figure 4C,D. (F) Fibre wall fraction plasticity was significant in only two species. Values declined from slightly positive to more negative, consistent with the model shown in Figure 4E,F. [Color figure can be viewed at wileyonlinelibrary.com]

Conduit density decreased plastically with site $P/E_{p}$ simultaneously with effects of species $P/E_{p}$ (both log‐transformed), but these did not interact, and plasticity did not differ between subgenera. Conduit density was offset 14.3 mm^−2^ lower in *Symphyomyrtus* ($R^{2}=0.71$; Figure 4C,D; Supporting Information S2: Table S3). Mean plasticity across sites was −16.5 mm^−2^, shallower than the effect of species $P/E_{p}$ (−93.8). Adding random effects did not improve models (species: *p* = 1; site: *p* = 0.22). Individually, no species had a significant relationship with log site $P/E_{p}$, but together, a negative common slope was supported (*p* = 0.03; $R^{2}=0.76$; Supporting Information S2: Table S2). Individual plasticities showed no trend with species $P/E_{p}$ (Figure 5E).

$A_{\mathrm{wall}}/A_{\mathrm{fib}}$ generally decreased plastically with site $P/E_{p}$, which interacted with species $P/E_{p}$ (*p* = 0.018). Plasticity did not differ by subgenus (*p* = 0.73) (Figure 4E,F). Overall, $R^{2}=0.82$. This model predicted differential plasticity. Plasticity was positive but near zero for *E. dumosa* (0.03 μm^2^ μm^−2^) and negative for other species, down to −0.11 in *regnans*. Plastic responses were shallower than relationships to species $P/E_{p}$ in *Symphyomyrtus*, ranging from −0.26 (Hattah) to −0.45 (Toolangi) μm^2^ μm^−2^. Site was not significant as a random effect (*p* = 0.87), but species was (*p* = 0.01), altering predictions in the same pattern as for $A_{\mathrm{wall}}/A_{\mathrm{fib}}$ versus species $P/E_{p}$. Individually, $A_{\mathrm{wall}}/A_{\mathrm{fib}}$ plasticity was negative and significant in 2 of 10 species and weak in 2 others. Slopes were not significantly different (*p* = 0.14; Supporting Information S2: Table S2), but a negative common slope was supported (*p* = 0.01). Individual slopes declined with species $P/E_{p}$ (Figure 5F).

### 3.3 Anatomical Basis of Hydraulic Conductivity Variation

$K_{x}$ increased with $K_{x,\mathrm{theo}}$ with offsets by site and species ($R^{2}=0.65$; Figure 6A–D; Supporting Information S2: Table S4). These data and all others under this header were log‐transformed. Most $K_{x,\mathrm{theo}}$ values were above observations. A common slope of 0.38 was observed. This model predicted that at a given $K_{x,\mathrm{theo}}$, $K_{x}$ increased roughly with species $P/E_{p}$ and increased in the order of site $P/E_{p}$.

**Figure 6:**
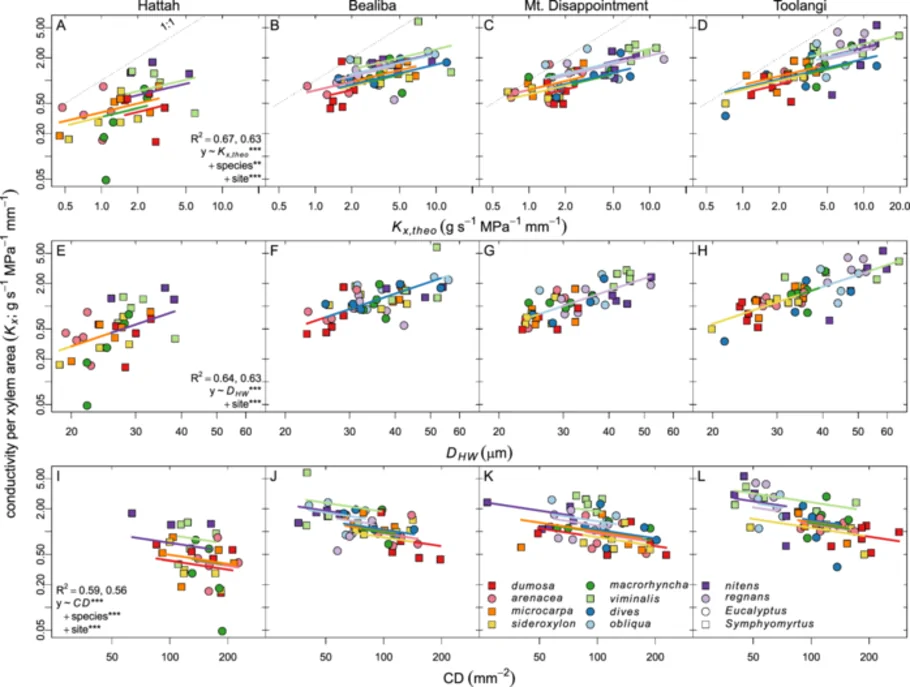
Hydraulic conductivity ($K_{x}$) variation explained by xylem anatomy (rows) plus site (columns) and species (point colours). For all panels, points are individual, uncorrected measurements to which regressions were fit. Text in the first column lists model details for each row, including multiple and adjusted $R^{2}$ values, model parameters and the significance of their removal. All numeric variables were log‐transformed. (A–D) Observed and theoretical hydraulic conductivity scaled positively at the same rate across sites but with site‐ and species‐specific offsets, predicting $K_{x}$ increased roughly with species $P/E_{p}$ (precipitation per pan evaporation) and in order of site $P/E_{p}$ at a given $K_{x,\mathrm{theo}}$. (E–H) Hydraulically weighted mean conduit diameter explained $K_{x,}$ arguably better than $K_{x,\mathrm{theo}}$. Slopes did not differ between sites and were offset by site only, yielding the same adjusted $R^{2}$ as $K_{x,\mathrm{theo}}$, but using fewer degrees of freedom. (I–L) $K_{x}$ declined with conduit density at the same rate across sites and with offsets by site and by species, such that at a given conduit density, $K_{x}$ tended to increase with species $P/E_{p}$ and increase in order of site $P/E_{p}$. [Color figure can be viewed at wileyonlinelibrary.com]

$K_{x}$ increased with conduit diameter with offsets between sites ($R^{2}=0.64$; Figure 6E–H). Intercepts increased in order of site $P/E_{p}$, and a common slope of 1.64 was found. $K_{x}$ declined with conduit density and with offsets between sites and species. At a given conduit density, $K_{x}$ increased in order of site $P/E_{p}$ and tended to be higher among species with higher $P/E_{p}$ (Figure 6I–L). Overall, $R^{2}=0.59$.

### 3.4 Anatomical Basis of Xylem + Pith Density Variation

$\rho_{x,p}$ increased with $A_{\mathrm{pith}}/A_{x,p}$ and $A_{\mathrm{wall}}/A_{x,p}$, which interacted with site (all *p* < 0.001; $R^{2}=0.68$; Figure 7A–D; Supporting Information S2: Tables S2 and S5). This regression suggested $\rho_{\mathrm{pith}}=0.357\;{\text{g cm}}^{-3}$ and $\rho_{\mathrm{wall}}$ was greatest at Hattah (0.862) and similar at other sites (0.744 to 0.775).

**Figure 7:**
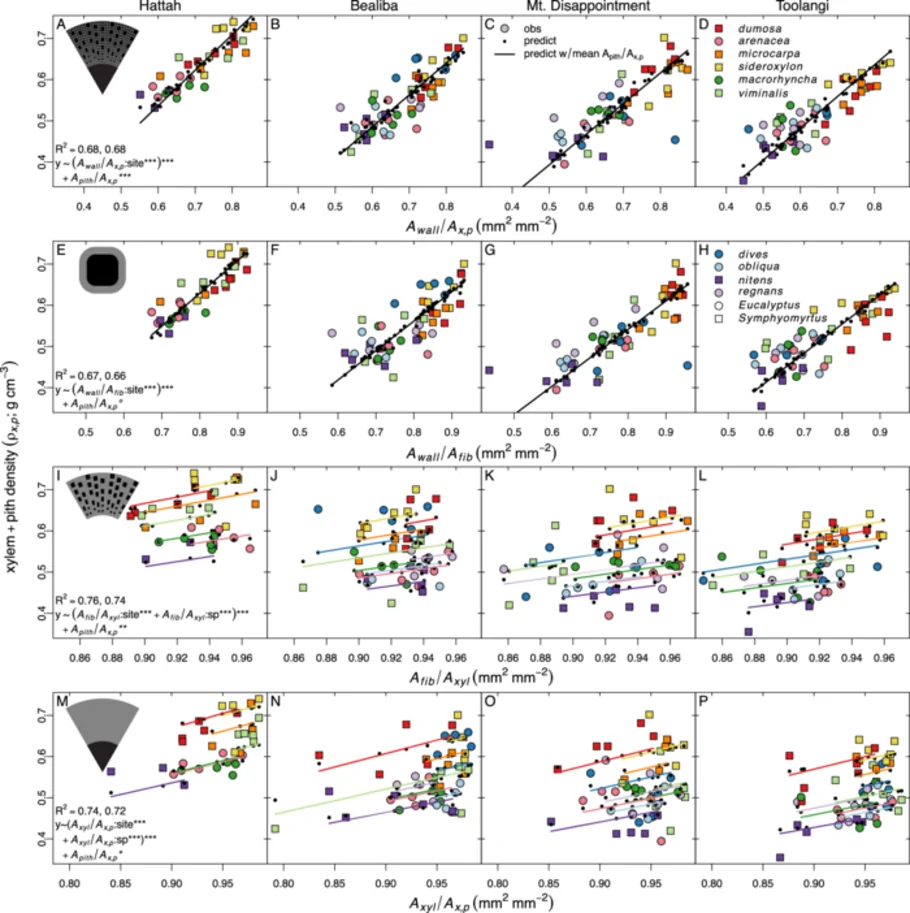
Xylem + pith density variation explained by xylem‐related fractions (rows), pith fraction, site (columns), and species (colours). For all panels, coloured points are individual measurements to which models were fit. Black points are model predictions using individual pith fraction measurements, while lines are predictions using group mean pith fractions. Diagrams depict each fraction as (grey area)/(grey + black area). Text in the first column lists model details for each row, including multiple and adjusted $R^{2}$ values, model parameters and the significance of their removal. (A–D) $\rho_{x,p}$ was well‐explained by $A_{\mathrm{wall}}/A_{x,p}$ and $A_{\mathrm{pith}}/A_{x,p}$. Site interacted with $A_{\mathrm{wall}}/A_{x,p}$, suggesting greater wall density at Hattah than at other sites. (E–H) Replacing $A_{\mathrm{wall}}/A_{x,p}$ with $A_{\mathrm{wall}}/A_{\mathrm{fib}}$ led to a very similar prediction of $\rho_{x,p}$, with site interactions predicting greater $\rho_{x,p}$ at Hattah than at other sites. (I–L) Replacing $A_{\mathrm{wall}}/A_{x,p}$ with $A_{\mathrm{fib}}/A_{\mathrm{xyl}}$ led to a more scattered relationship within sites, but some of this scatter could be explained by $A_{\mathrm{fib}}/A_{\mathrm{xyl}}$ interacting with species in addition to site. Lower $P/E_{p}$ species tended to have larger interaction coefficients. (M–P) $A_{\mathrm{xyl}}/A_{x,p}$ was a similar predictor as $A_{\mathrm{fib}}/A_{\mathrm{xyl}}$, with a more scattered relationship within sites that could be explained by $A_{\mathrm{xyl}}/A_{x,p}$ interacting with species in addition to site. Interaction coefficients suggested that xylem density decreased across sites as site $P/E_{p}$ (precipitation per pan evaporation) increased, and within sites, xylem density decreased as species $P/E_{p}$ increased. [Color figure can be viewed at wileyonlinelibrary.com]

Replacing $A_{\mathrm{wall}}/A_{x,p}$ in the regression with its components (Equation 4) showed that $A_{\mathrm{wall}}/A_{\mathrm{fib}}$ predicted $\rho_{x,p}$ similarly to $A_{\mathrm{wall}}/A_{x,p}$. Site interacted with $A_{\mathrm{wall}}/A_{\mathrm{fib}}$ while $A_{\mathrm{pith}}/A_{x,p}$ was a weak additional predictor ($R^{2}=0.67$; Figure 7E–H). Interaction coefficients were greatest at Hattah (0.798) and similar at other sites (0.690 to 0.715). $A_{\mathrm{fib}}/A_{\mathrm{xyl}}$ predicted $\rho_{x,p}$ well when interacting with site and species and added to $A_{\mathrm{pith}}/A_{x,p}$ ($R^{2}=0.76$; Figure 7I–L). Site coefficients decreased in order as site $P/E_{p}$ increased. Species coefficients were greatest among *Symphyomyrtus* species from low $P/E_{p}$ locations and tended to decline as species $P/E_{p}$ increased (Supporting Information S2: Table S5). Lastly, $A_{\mathrm{xyl}}/A_{x,p}$ predicted $\rho_{x,p}$ well when interacting with site and species and added to $A_{\mathrm{pith}}/A_{x,p}$ ($R^{2}=0.74$; Figure 7M–P). Coefficients suggested that $\rho_{\mathrm{pith}}=0.239\;g\;{\text{cm}}^{-3}$ and $\rho_{\mathrm{xyl}}$ ranged from 0.451 g cm^−3^ (*E. nitens* at Toolangi) to 0.728 (*E. sideroxylon* at Hattah). Across sites, $\rho_{\mathrm{xyl}}$ decreased in order of site $P/E_{p}$, while across species, xylem density roughly tended to decrease with species $P/E_{p}$.

### 3.5 Trait–Trait Correlations

Log‐transformed ${\dot{K}}_{x}$ declined with ${\dot{\rho }}_{x,p}$ ($R^{2}=0.41$) and increased with TLP ($R^{2}=0.44$). Neither ${\dot{\rho }}_{x,p}$ nor TLP explained ${\dot{K}}_{x}$ variation left unexplained by site $P/E_{p}$ and species $P/E_{p}$ (i.e., Figure 3A). ${\dot{\rho }}_{x,p}$ could replace some explanatory value of species $P/E_{p}$, while TLP could replace some explanatory value of site $P/E_{p}$. Both replacements reduced model fit from $R^{2}=0.72$ to 0.59 but remained significant: *p* = 0.002 (${\dot{\rho }}_{x,p}$) and *p* = 0.001 (TLP). ${\dot{\rho }}_{x,p}$ best explained ${\dot{K}}_{x}$ when allowing species‐specific offsets (*p* = 0.028) and slopes (*p* = 0.033; $R^{2}=0.89$; Figure 8A; Supporting Information S2: Table S6). These individual slopes and intercepts roughly separated species into two density groups, showing that species could have similar ${\dot{K}}_{x}$ with contrasting densities (Figure 8A). TLP best explained ${\dot{K}}_{x}$ when allowing site‐specific offsets (*p* = 0.021; $R^{2}=0.61$; Figure 8B). This model predicted that at a given TLP, ${\dot{K}}_{x}$ was lowest at Hattah and highest at Toolangi, but Bealiba and Mt. Disappointment did not appear in order of site $P/E_{p}$.

**Figure 8:**
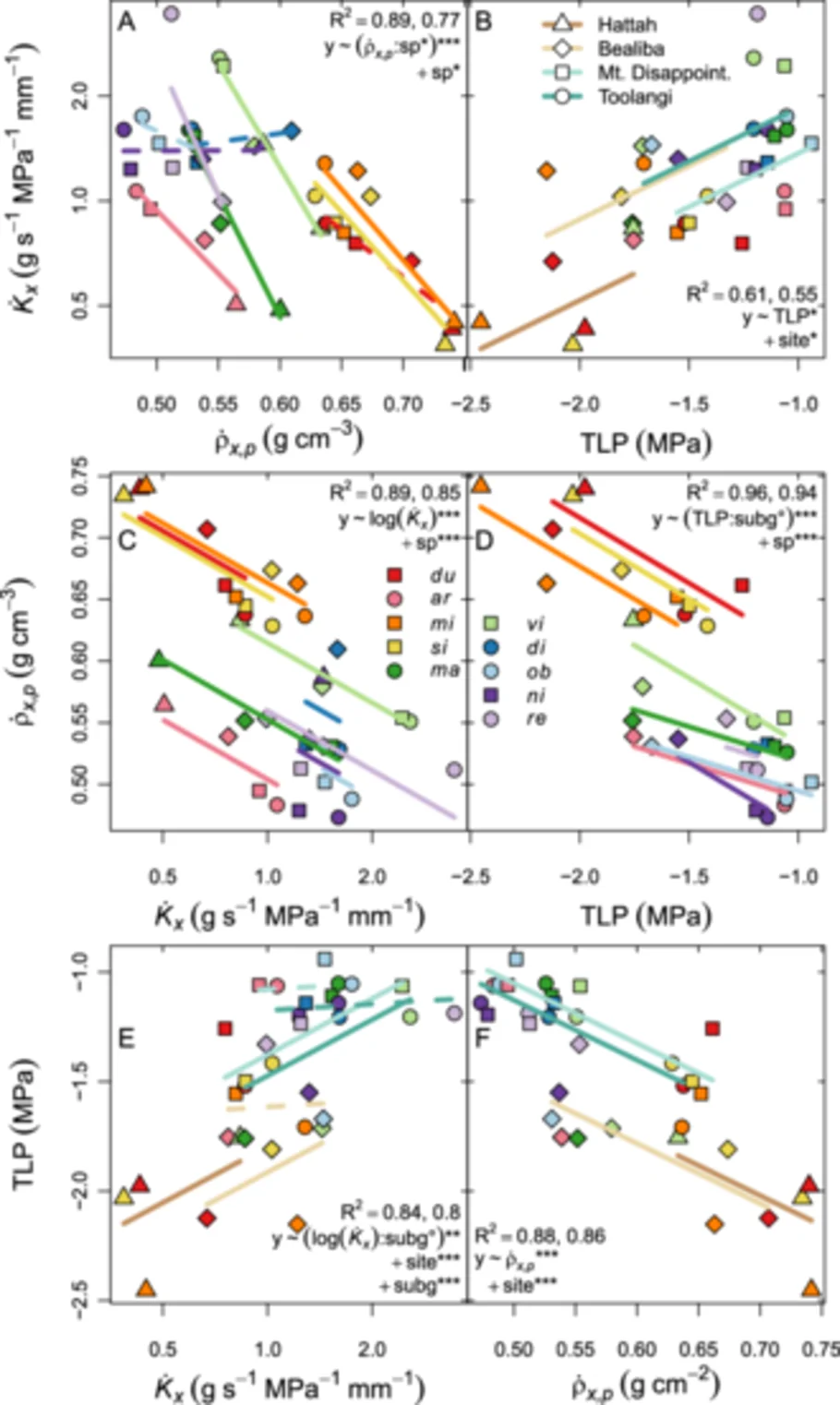
Trait–trait relationships for hydraulic conductivity, xylem + pith density and leaf turgor loss point. All panels: Points are means at each site*species combination. Before averaging, density and hydraulic conductivity were ‘corrected’ for sample diameter variation. The regressions used log‐transformed hydraulic conductivity. Text in the lower corners shows multiple and adjusted $R^{2}$ values and model parameters with their significance. (A) Hydraulic conductivity declined with xylem + pith density and was well explained by the interaction between density and species. Six of 10 species had significant slopes, indicated by solid lines. (B) Hydraulic conductivity increased with TLP. Sites were offset, with Hattah having the lowest ${\dot{K}}_{x}$ at a given TLP. (C and D) ${\dot{\rho }}_{x,p}$ declined with ${\dot{K}}_{x}$ and TLP. Variation was further explained by species‐specific offsets. (E) TLP increased with ${\dot{K}}_{x}$ with offsets separating sites (coloured lines) and subgenera (small black points). At a given ${\dot{\rho }}_{x,p}$, wetter sites Mt. Disappointment and Toolangi had less negative TLP than drier sites Bealiba and Hattah, while subgenus *Eucalyptus* had less negative TLP than *Symphyomyrtus*. (F) TLP also declined with ${\dot{\rho }}_{x,p}$, with offsets between sites but not between subgenera. [Color figure can be viewed at wileyonlinelibrary.com]

${\dot{\rho }}_{x,p}$ declined with ${\dot{K}}_{x}$ ($R^{2}=0.41$) and TLP ($R^{2}=0.65$). Neither ${\dot{K}}_{x}$ nor TLP explained ${\dot{\rho }}_{x,p}$ variation left unexplained by site $P/E_{p}$, species $P/E_{p}$ and subgenus (i.e., Figure 3B,C). ${\dot{K}}_{x}$ or TLP could replace some explanatory value of site $P/E_{p}$, reducing model fit from $R^{2}=0.95$ to 0.88 (${\dot{K}}_{x}$) or 0.92 (TLP). ${\dot{\rho }}_{x,p}$ was best explained when allowing species‐specific offsets with ${\dot{K}}_{x}$ (*p* < 0.001; $R^{2}=0.89$) or TLP (*p* < 0.001; $R^{2}=0.95$; Figure 8C,D; Supporting Information S2: Table S6).

TLP increased with ${\dot{K}}_{x}$ ($R^{2}=0.44$) and ${\dot{\rho }}_{x,p}$ ($R^{2}=0.65$). Neither ${\dot{K}}_{x}$ nor ${\dot{\rho }}_{x,p}$ explained TLP variation left unexplained by site $P/E_{p}$, species $P/E_{p}$ and subgenus (i.e., Figure 3D,E). ${\dot{K}}_{x}$ could replace some explanatory value of species $P/E_{p}$ and subgenus, reducing model fit from $R^{2}=0.82$ to 0.76. ${\dot{\rho }}_{x,p}$ offered slightly more explanation of TLP than species $P/E_{p}$ and subgenus, increasing $R^{2}$ from 0.82 to 0.83 while reducing degrees of freedom used. ${\dot{K}}_{x}$ best explained TLP when allowing offsets for site (*p* < 0.001) and subgenus (*p* = 0.003; $R^{2}=0.82$; Figure 8E; Supporting Information S2: Table S6). This model separated sites into two groups, with the two drier sites having, on average, 0.46 MPa more negative TLP at a given ${\dot{K}}_{x}$ than wetter sites. On top of these site differences, subgenus *Symphyomyrtus* had 0.22 MPa more negative TLP at a given ${\dot{K}}_{x}$ than *Eucalyptus*. ${\dot{\rho }}_{x,p}$ best explained TLP when allowing offsets for site (*p* < 0.001; $R^{2}=0.88$; Figure 8F), which separated sites similarly to ${\dot{K}}_{x}$.

## 4 Discussion

By growing a set of 10 *Eucalyptus* species – stratified by subgenus and native mean $P/E_{p}$ – across 4 common gardens that span a $P/E_{p}$ gradient, we documented substantial variation in 6 key hydraulic traits and leveraged this experimental framework to rigorously separate trait variation due to plasticity versus due to genetically fixed variation. Traits generally varied with $P/E_{p}$ in expected directions (H1 and H2) and with genetically fixed variation outweighing plasticity. Directions of trait‐environment covariation often agreed with prior studies on *Eucalyptus* (Pfautsch et al. 2016; Bourne et al. 2017; Pan et al. 2025; Britton et al. 2026), but our experimental design removes ambiguity about causes of variation. Exceptions to H1 came from subgenus *Eucalyptus* species that sometimes had uniform trait values despite originating from a wide range of climates similar to *Symphyomyrtus* study species (H6). This finding deserves further study on more species and more traits to determine its prevalence and implications. In addition to quantifying plastic variation, we found some evidence for differential plasticity between species – a metric not often tested in plasticity studies (*but see* Medeiros and Pockman 2014; Guha et al. 2018) – where species from wetter origins had greater plasticity in three traits (H3). Determining how and why plasticity varies between taxa will improve our understanding of how they may differentially respond to environmental perturbation. We found that, anatomically, xylem + pith density was strongly driven by fibre wall fraction while hydraulic conductivity was unsurprisingly driven by conduit diameter (H4). Meanwhile, hydraulic conductivity, xylem + pith density and TLP were correlated, but their covariation could not aid or replace the explanatory value of $P/E_{p}$ (H5). In the remainder of the discussion, we explore possible implications of our findings.

### 4.1 Implications for Whole Plant Water Transport

Hydraulic conductivity increased with site $P/E_{p}$ and species $P/E_{p}$ largely as we predicted. What are the potential impacts of these differences across sites and across species? That hydraulic conductivity can influence stomatal conductance ($g_{s}$) and leaf water potential ($\psi_{\mathrm{leaf}}$) can be envisaged via the equation: $\text{DF}g=\frac{k}{A_{\mathrm{leaf}}}(\psi_{\mathrm{soil}}-\psi_{\mathrm{leaf}})$, where DF is the driving force for transpiration (mol mol^−1^; von Caemmerer and Farquhar 1981), *g* is total diffusive conductance to water vapour (mol m^−^^2^ s^−^^1^; a function of $g_{s}$), k is hydraulic conductance of the soil to leaf pathway (mol MPa^−^^1^ s^−^^1^; a function of $K_{x}$), $A_{\mathrm{leaf}}$ is total leaf area (m^2^) and ψ are water potentials of soil and leaf (MPa). If k increases with site $P/E_{p}$, then plasticity in one or more parameters is needed to accommodate this increase. Considering parameters singly, one potential scenario is that greater k supports higher g with constant $\psi_{\mathrm{leaf}}$. Thus, all increases in k would translate to increased gas exchange. A contrasting scenario is that greater k supports less negative $\psi_{\mathrm{leaf}}$ with constant g. This scenario realizes increased k as increased safety from the detrimental effects of low $\psi_{\mathrm{leaf}}$. We recently showed that both $g_{s}$ and midday $\psi_{\mathrm{leaf}}$, increase with site $P/E_{p}$ (Smith et al. 2023), suggesting a compromise between the two hypothetical scenarios. However, we also showed that predawn $\psi_{\mathrm{leaf}}$ (a proxy for $\psi_{\mathrm{soil}}$) increased with site $P/E_{p}$ (Smith et al. 2023), in a way that maintains constant $\psi_{\mathrm{soil}}-\psi_{\mathrm{leaf}}$. Together, these suggest that increasing k translates nearly directly into increased g while increasing $\psi_{\mathrm{soil}}$ eases $\psi_{\mathrm{leaf}}$. Allowing DF to vary as well, we expect DF to decrease with site $P/E_{p}$. Doing so in this scenario requires g to increase faster than it would under constant DF, further increasing potential photosynthetic gain. These hypothetical scenarios rely on several assumptions, particularly about k and $A_{\mathrm{leaf}}$. Further studies examining how k and $A_{\mathrm{leaf}}$ plastically change together in response to relative moisture supply would help to elucidate how plastic changes to hydraulic architecture translate into changes in photosynthetic gas exchange.

### 4.2 Plastic Versus Genetically Fixed Effects

Traits often shifted plastically with site $P/E_{p}$ less than they shifted with species $P/E_{p}$ (Figures 3 and 4). If plastic and fixed effects are equal, then reciprocal transplants between environments would develop equivalent traits, intermediate to traits in their native environments. Stronger fixed effects would pull the traits of transplants back towards their native values, while stronger plasticity would push traits towards native traits in the new environment. Supposing that species' traits are optimal in their native environment, stronger fixed effects would tend to maintain niche differentiation better. Full plasticity with no fixed effects would erase niche differentiation, and species would lack a competitive edge in their native environment. It is then notable that we found fixed effects often stronger than plasticity. An obvious exception was that, in subgenus *Eucalyptus*, ${\dot{\rho }}_{x,p}$, TLP, and $A_{\mathrm{wall}}/A_{\mathrm{fib}}$ had nearly zero fixed effects, leaving essentially only plasticity driving trait differences across the study. While plasticity dominated in these subgenus*trait combinations, plasticity of ${\dot{\rho }}_{x,p}$ and $A_{\mathrm{wall}}/A_{\mathrm{fib}}$ was still weak compared to fixed effects in subgenus *Symphyomyrtus*, thus reducing the apparent importance of plasticity dominance. By contrast, TLP had nearly equal plasticity and fixed effects in subgenus *Symphyomyrtus*. Given the regression surfaces in Figures 3 and 4, one can find directions of steepest ascent across the $P/E_{p}$ landscape and ask if the directions point more towards plasticity or fixed effects (Supporting Information S1: Figure S5). We did so and scaled values between 0 (fully plastic) and 1 (fully fixed). TLP in *Symphyomyrtus* averaged 0.52. Ignoring the exceptions that lacked fixed effects, other traits averaged more toward fixed. Their means were 0.66 (${\dot{K}}_{x}$), 0.83 (${\dot{\rho }}_{x,p}$ and ${\dot{D}}_{HW}$), 0.88 ($\dot{\mathrm{CD}}$) and 0.93 ($A_{\mathrm{wall}}/A_{\mathrm{fib}}$). Of our study traits, TLP should be the most labile – due to its dependence on cellular osmotica compared to other study traits that have more structural basis – so its nearly equivalent plasticity and fixed effects may be expected. While means usually favoured fixed effects, some combinations of site $P/E_{p}$ and species $P/E_{p}$ favoured plasticity in ${\dot{K}}_{x}$ and ${\dot{D}}_{HW}$. These combinations occurred for high $P/E_{p}$ species grown at low $P/E_{p}$ sites (Supporting Information S1: Figure S5A). A possible explanation is that decreasing site $P/E_{p}$ reduced turgor pressure for conduit expansion, overriding genetic controls more so than at other site*species combinations.

### 4.3 Anatomical Explanation of Xylem Density

We found a good anatomical explanation for variation in $\rho_{x,p}$ (H4) across individual samples, with $R^{2}$ values between 0.67 and 0.74, using various anatomical fractions (see Figure 7). For all these models, site was a significant term, allowing for the models to account for differences in $\rho_{x,p}$ across sites, despite equivalent anatomy. Models with $A_{\mathrm{wall}}/A_{x,p}$ or $A_{\mathrm{wall}}/A_{\mathrm{fib}}$ were not improved significantly when these fractions interacted with species (*p* < 0.13). However, models with $A_{\mathrm{fib}}/A_{\mathrm{xyl}}$ or $A_{\mathrm{xyl}}/A_{x,p}$ did benefit from species interactions. Taken together, these patterns suggest that $A_{\mathrm{wall}}/A_{\mathrm{fib}}$ was the most proximal cause of $\rho_{x,p}$ variation between species. Fractions $A_{\mathrm{fib}}/A_{\mathrm{xyl}}$ and $A_{\mathrm{xyl}}/A_{x,p}$ do not account for fibre wall dimensions, and so their regressions benefit from species interaction to take the explanatory place of differences in $A_{\mathrm{wall}}/A_{\mathrm{fib}}$ between species. Site‐dependent differences in $\rho_{x,p}$ could be due to different levels of extractable compounds, particularly more compounds at Hattah, to give them a higher density. In other *Eucalyptus* species, Searson et al. (2004) found water limitation could increase density by increasing extractable compounds. Removing extractable compounds negated this effect of water limitation on density in two of their three species, including *E. sideroxylon*. We expect that extracting compounds from our samples would similarly negate differences between sites. Preliminary data from a subset of samples used in this study showed that extraction reduced $\rho_{x,p}$ between 19% and 26% (site means), but significant differences between sites remained (DDS unpublished data), leaving site differences in $\rho_{x,p}$ not fully explained. Our regressions suggested site‐dependent hydrated wall densities between 0.744 and 0.862 g cm^−3^. These values are among or below the minimum values found by Kellogg and Wangaard (1969) in 13 other hardwoods, using generally a similar methodology. Differences between the literature values and ours may be due to small methodological differences. Our consistent treatment of samples across sites and species suggests that relative differences are robust even if accuracy is not absolute.

### 4.4 Subgeneric Contrasts

Subgenera *Eucalyptus* and *Symphyomyrtus* often differed in their trait relationships, in opposition to our expectation (H6). H1 was consistently supported by subgenus *Symphyomyrtus* but only partly supported by subgenus *Eucalyptus*. In subgenus *Eucalyptus*, ${\dot{\rho }}_{x,p}$, $A_{\mathrm{wall}}{/A}_{\mathrm{fib}}$ and TLP showed no significant trend with species $P/E_{p}$. Trends in *Symphyomyrtus* suggested greater tolerance of lower water potentials with decreasing $P/E_{p}$, as we expected based on subgenus distributions. Trait values in all subgenus *Eucalyptus* study species aligned closer to *Symphyomyrtus* species that have high native $P/E_{p}$, suggestive of consistently low tolerance of low water potentials. This lack of a trend is despite subgenus *Eucalyptus* study species spanning from the mallee (*E. arenacea*) to tall wet sclerophyll forests (*E. regnans*). Across the entire genus, subgenus *Eucalyptus* species are generally absent from the driest sites, while large numbers of *Symphyomyrtus* species are found in arid and semi‐arid mallee, as well as woodlands and wet forests (Thornhill et al. 2019), Among our study species, maximum photosynthetic rates measured by Salvi et al. (2021) under greenhouse conditions fall to zero at leaf water potentials of −2.8 to −4.5 MPa in subgenus *Eucalyptus*, while the same, potentially fatal condition occurs at leaf water potentials of −3.5 to −6.5 MPa in *Symphyomyrtus*. A lack of traits for tolerating low water potentials may suggest an aversion to them. Avoiding (rather than tolerating) low tissue water potentials is a valid strategy for plants to survive in environments with low moisture availability, raising the possibility that species in subgenus *Eucalyptus* exhibit a more conservative (isohydric) stomatal control. Salvi et al. (2022) recently showed that, in our 10 study species, hydroscape area (a measure of anisohydry) was greatest in 4 *Symphyomyrtus* species, which also had the most negative TLP and the lowest hydraulic and photosynthetic sensitivities to low water potential, suggesting that several dominant *Symphyomyrtus* species in Victoria can maintain positive carbon balance at lower water potentials than the dominant species of subgenus *Eucalyptus*. Our study examined only 10 of the hundreds of *Eucalyptus* species, so we cannot draw broad conclusions with certainty about trait differences between subgenera (Adams 2000). Differential adaptation to water stress associated with drought and temperature was also recently quantified in 14 species in Tasmania, with (1) leaf embolism resistance declining significantly as minimum monthly temperatures rise in subgenus *Symphyomyrtus* but not subgenus *Eucalyptus*; and (2) minimum stomatal conductance ($g_{\min}$) decreasing in similar fashion in both subgenera along a gradient from cold/wet habitats to warm/dry habitats (Hartill et al. 2023). We note, however, that in all but one case for the second regression, species of subgenus *Eucalyptus* have lower $g_{\min}$ values than those of *Symphyomyrtus* at the same position along the climatic gradient, and that – except at the warmest/driest conditions – species of subgenus *Eucalyptus* have lower embolism resistance than species of *Symphyomyrtus* at the same point along the climatic gradient. Hartill et al. (2023) thus also found some support for members of subgenus *Symphyomyrtus* often being better adapted to drier conditions than subgenus *Eucalyptus*.

### 4.5 Responses to Climate Change

Southeastern Australia is generally predicted to become drier and warmer as climate change progresses, which should drive down $P/E_{p}$. In particular, a 2090 scenario with CO_2_ stabilizing around 540 ppm predicts median changes around −16% for annual rainfall and +7% for potential evapotranspiration (used as a proxy here for pan evaporation; CSIRO and Bureau of Meteorology 2015; Dowdy et al. 2015; Ekström et al. 2015; Timbal et al. 2015). Together, these suggest a 21% decrease in $P/E_{p}$. Our first expectation is that species should shift their distributions geographically southeastward so that their distributions along the $P/E_{p}$ gradient remain more or less fixed. Smith et al. (2023) showed that the study species had a ‘home field’ advantage in height growth. This pattern of adaptive cross‐over helps account for species distributions along the $P/E_{p}$ gradient and takes into account plasticity. Changing climates will undoubtedly disrupt current correlations between temperature and $P/E_{p}$; thermal effects *per se*, as well as impacts of differences in soils and other organisms, are likely further influences on species distributions.

### 4.6 Mechanisms of Plasticity

Despite some trait relationships varying by subgenus within sites, we found no evidence that plasticity differed between subgenera, which is an interesting contrast, suggesting an equivalent mechanism for trait plasticity in both subgenera. We can consider the possibility that the plasticity of xylem traits is passively due to water potential influencing cellular expansion. If lower water potential proportionally reduces all cell volumes, then we should expect specific allometries between different measures in our study. If xylem volume changes are realized through changes in cell diameter and not cell length, then we should expect $\rho_{x,p}$, CD and $A_{\mathrm{wall}}/A_{\mathrm{fib}}$ to scale isometrically with each other. In accord, we did find that ${\dot{\rho }}_{x,p}$ and $\dot{CD}$ both decrease linearly as site $P/E_{p}$ increases (Figure 4A–D). However, $A_{\mathrm{wall}}/A_{\mathrm{fib}}$ variably decreases or increases as a function of species $P/E_{p}$ (Figure 4E,F), indicating a disconnect from simple volume changes. Further, we found that some $\rho_{x,p}$ plasticity was not explained by anatomy (Figure 7). If plasticity is driven by cellular volume changes, we would also expect that conduit diameter and conduit density scale in a way that preserves the fractional area occupied by conduits. That is, conduit density should scale with conduit diameter^−^^2^. Using all the data (rather than site*species means), we divided conduit densities by conduit diameter^−^^2^, yielding fractional area occupied by conduits, which correlated positively with site $P/E_{p}$, indicating that conduits occupy less fractional area at drier sites. This trend is suggestive that as conduit diameter decreases, fewer conduits are produced, thus arguing against cellular volume changes solely driving plasticity.

## 5 Conclusions

Understanding how plant traits have evolved to succeed in specific environments and how those traits change to suit temporal and spatial environmental variation is critical to predicting species distributions now and in future. Here, we have shown considerable differences in hydraulic traits between *Eucalyptus* species native to areas with contrasting relative moisture supplies. We show that most of these traits change plastically with the environment. Such changes vary in extent with trait and species. Many observed inter‐ and intra‐specific differences appear adaptive for the purposes of adjusting to water availability and acquiring carbon. Further questions are raised about how these differences are realized and their mechanistic impacts on species success at a given location. The use of multiple common gardens is a key approach to informing species distributions.

## Supporting information


Supporting File 1

Supporting File 2

## Data Availability

The data that support the findings of this study are available from the corresponding author upon reasonable request.
