# Supplementary material for: Diversity and Plasticity of Hydraulic Traits Across a Relative Moisture Supply Gradient: Results From Multiple Common Gardens
Source: Plant Cell Environ. 2026 Sep 16;49(12):10207–24. doi: 10.1111/pce.70890 (PMC13725259; doi:10.1111/pce.70890)
Supplement: Supplementary file 1 [file PCE-49-10207-s002.pdf]

Supplement to:

Diversity and plasticity of hydraulic traits across a relative moisture supply gradient: Results from multiple common gardens

Duncan D. Smith[1,2], Thomas J. Givnish[1], Mark A. Adams[3], Katherine A. McCulloh[1]

[1]Department of Botany, University of Wisconsin - Madison, Madison, WI, 53706, USA
[2]School of Ecosystem and Forest Sciences, University of Melbourne, Creswick, Victoria 3363 Australia
[3]School of Life and Environmental Sciences, University of Sydney, NSW 2006, Australia

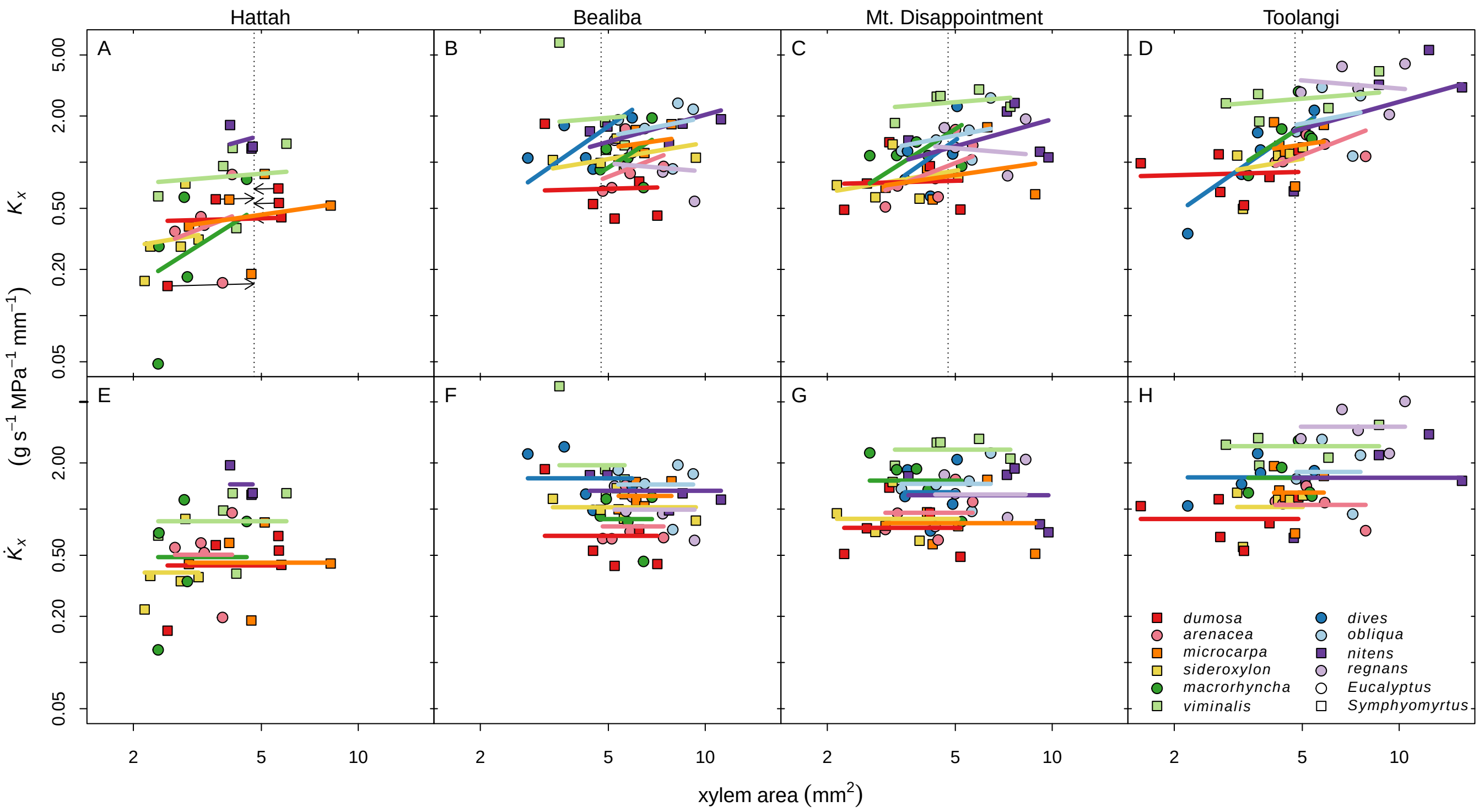

Figure S1: Illustration of how K_x (hydraulic conductivity per xylem area) was adjusted to remove residual influences of xylem area (A_{xyl}). Each point represents one stem. **A-D**: Xylem areas varied between sites and species and K_x often correlated with xylem area, as shown by regression lines. For each species we fit the data as $\log(K_x) \sim \log(A_x) + site$ (i.e., slopes differ between species, within species slopes are the same across sites, within species intercepts differ across sites). Using the slope and residual of each point, we estimated K_x at the grand median A_{xyl} (vertical lines). Arrows in panel **A** indicate how K_x values were adjusted for *E. dumosa*. **E-H**: Rescaled K_x ($\dot{K}_x$) is not influenced by xylem area, as shown by regression lines, allowing the influences of species and site to be assessed without confounding effects of xylem area. We standardized xylem+pith density by a similar process.

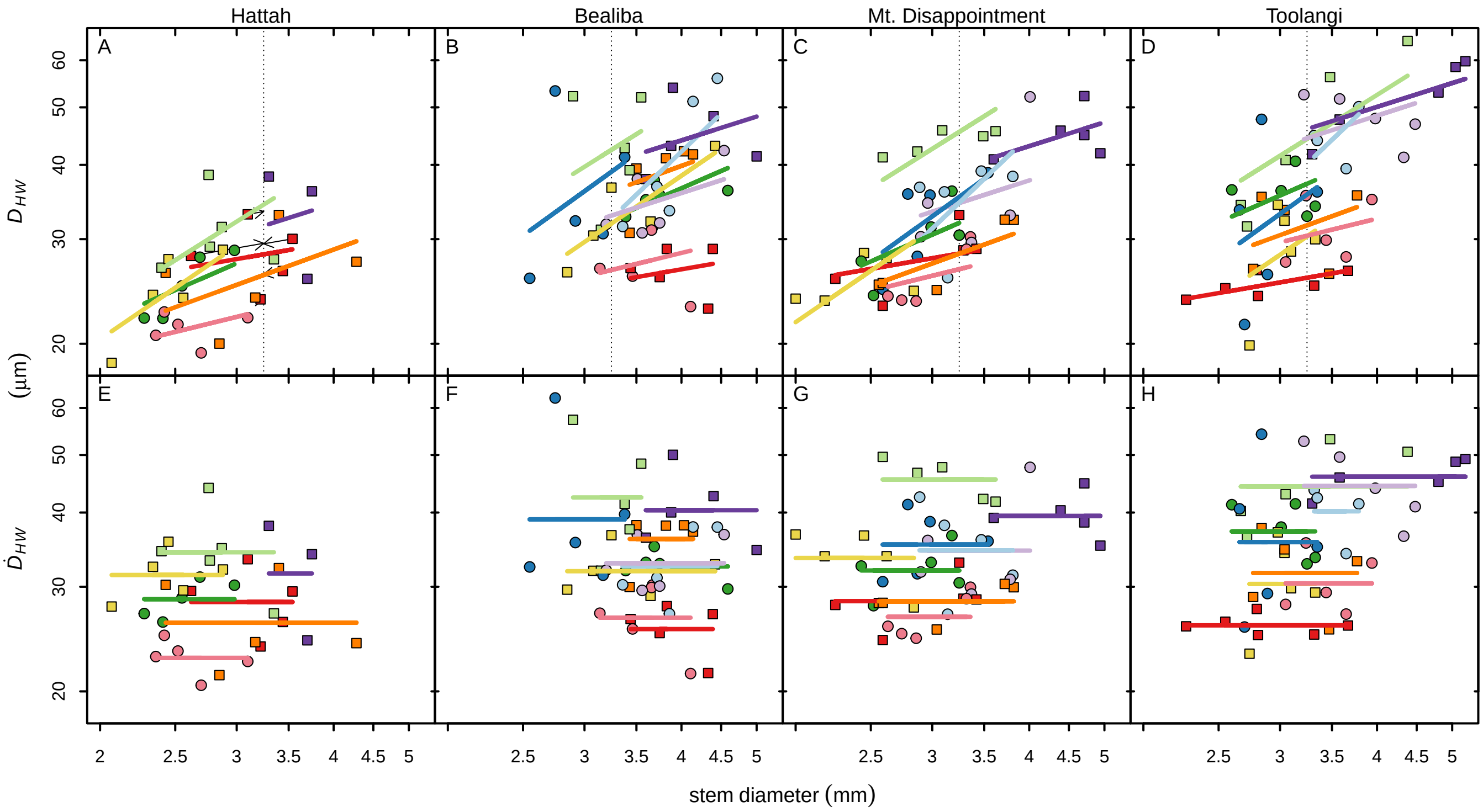

Figure S2: Illustration of how D_{HW} (hydraulically-weighted conduit diameter) was adjusted to remove influences of stem diameter (D_{stem}). Each point represents one stem. Symbol key in Fig. S1. **A-D**: Stem diameters varied between sites and species and D_{HW} often correlated with stem diameter, as shown by regression lines. For each species we fit the data as $\log(D_{HW}) \sim \log(\mathrm{D_{stem}}) + site$ (i.e., slopes differ between species, within species slopes are the same across sites, within species intercepts differ across sites). Using the slope and residual of each point, we estimated D_{HW} at the grand median D_{stem}(vertical lines). Arrows in panel **A** indicate how D_{HW} values were adjusted for *E. dumosa*. **E-H**: Rescaled D_{HW} ($\dot{D}_{HW}$) is not influenced by stem diameter, as shown by regression lines, allowing the influences of species and site to be assessed without confounding effects of stem diameter.

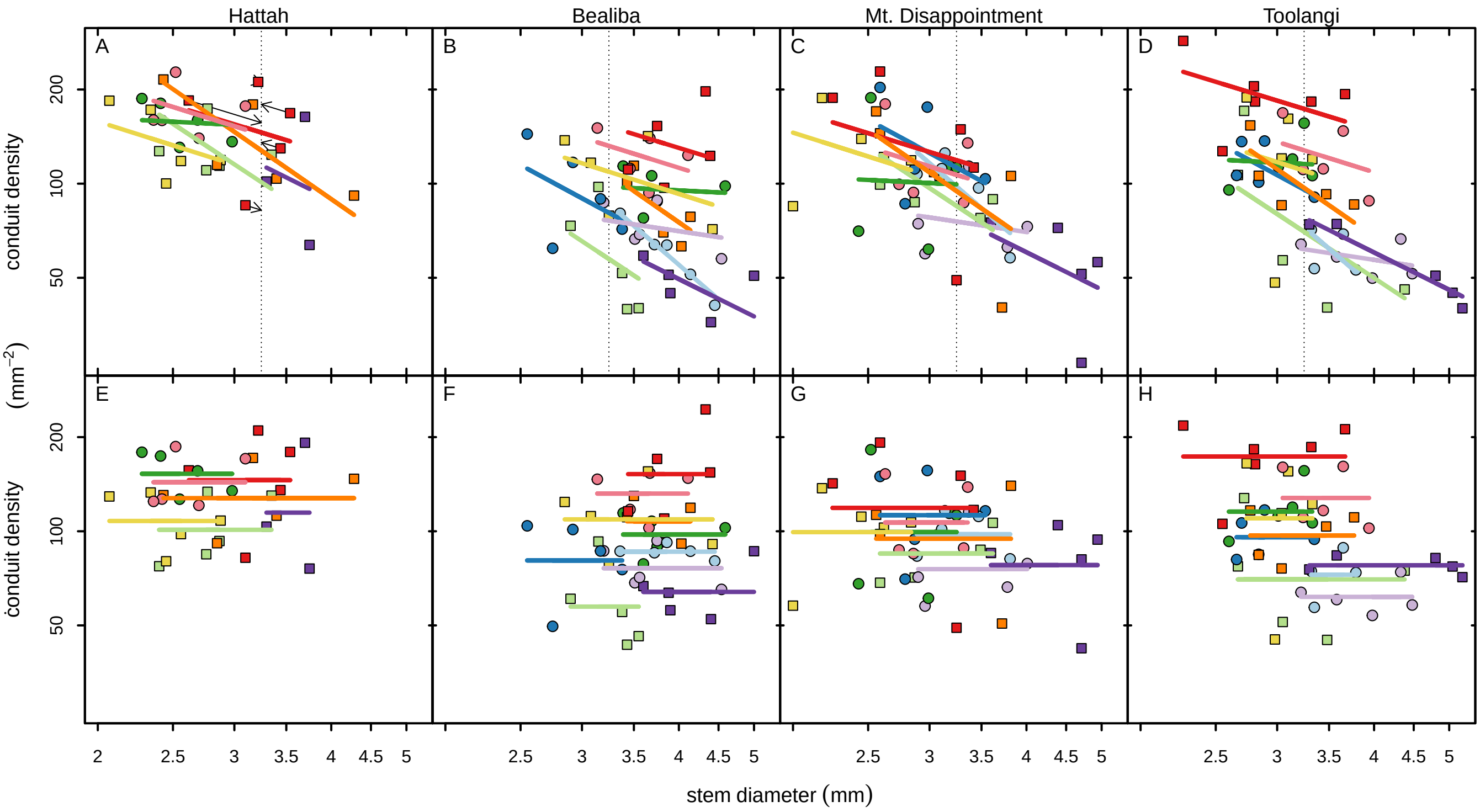

Figure S3: Illustration of how conduit density was adjusted to remove influences of stem diameter (D_{stem}). Each point represents one stem. Symbol key in Fig. S1. **A-D**: Stem diameters varied between sites and species and conduit density often correlated with stem diameter, as shown by regression lines. For each species we fit the data as log(conduit density) $\sim$ $\log(\mathrm{D_{stem}})$ + *site* (i.e., slopes differ between species, within species slopes are the same across sites, within species intercepts differ across sites). Using the slope and residual of each point, we estimated conduit density at the grand median D_{stem}(vertical lines). Arrows in panel **A** indicate how conduit density values were adjusted for *E. dumosa*. **E-H**: Rescaled conduit density ($\dot{C}D$) is not influenced by stem diameter, as shown by regression lines, allowing the influences of species and site to be assessed without confounding effects of stem diameter.

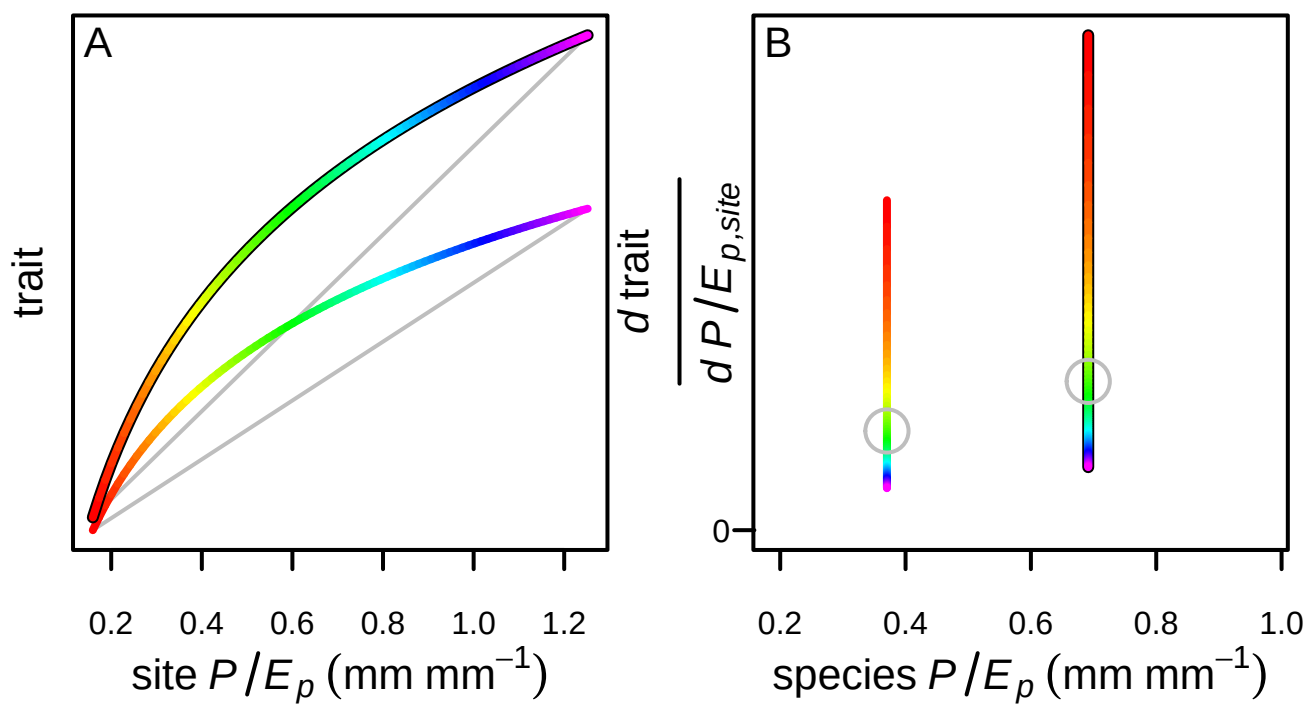

Figure S4: Illustration how regressions fit to trait vs log-transformed site P/E_p suggest site-dependent plasticity. For two hypothetical species, trait increased curvilinearly with site P/E_p (precipitation per pan evaporation), following the equation $trait = a \log(P/E_{p,site}) + b$, where a and b are species-specific coefficients (A). Plasticity ($\frac{d\text{trait}}{dP/E_{p,site}}$) equals $\frac{a}{P/E_{p,site}}$, meaning plasticity becomes shallower as site P/E_p increases, as shown by vertical lines in panel B. We then calculated mean plasticity of each species as the slope of the chord connecting trait predictions at minimum and maximum site P/E_p (grey lines in A, grey circles in B).

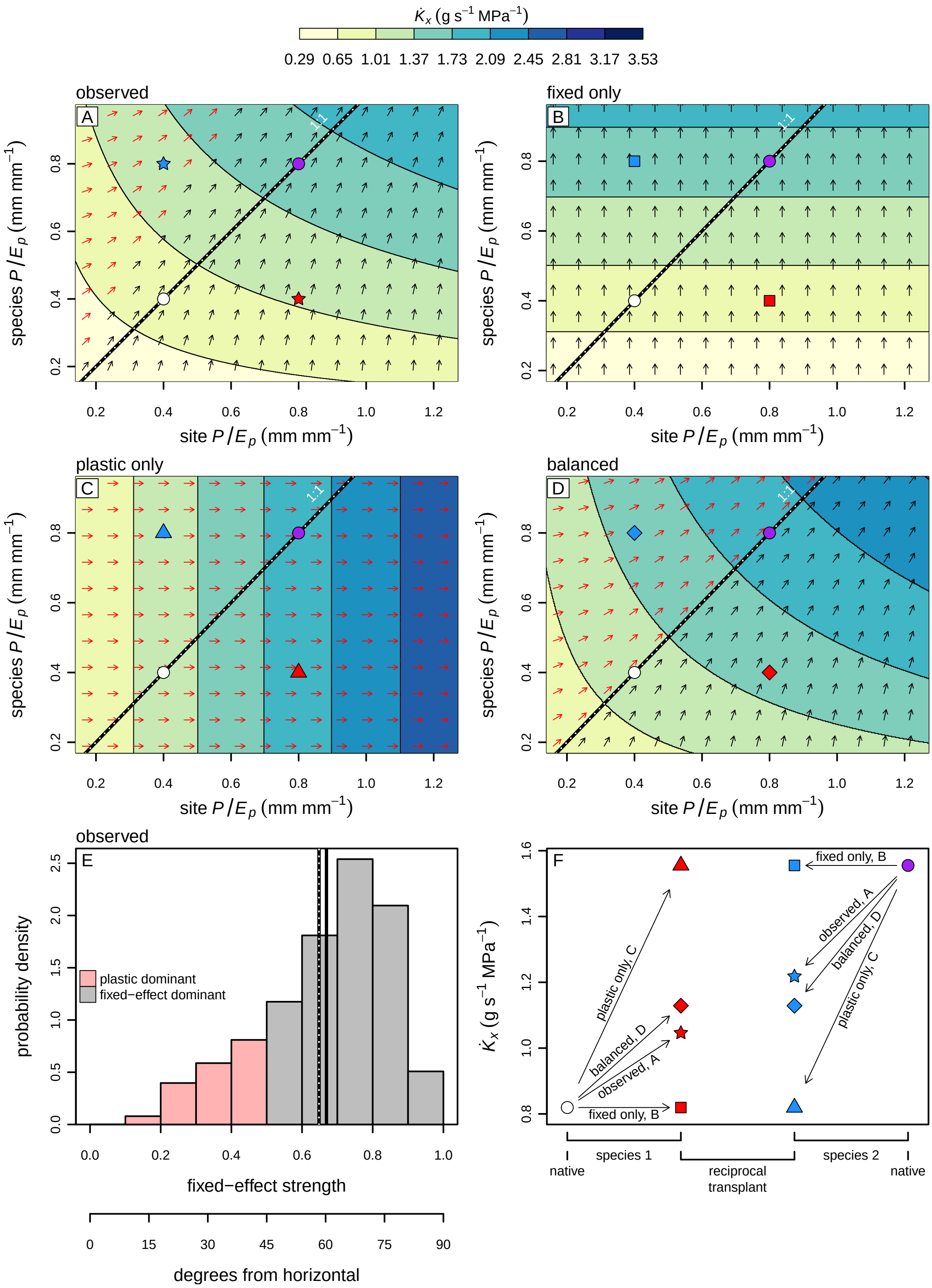

Figure S5: caption on following page

Figure S5: Contour plots that predict a trait from site P/E_p (precipitation per pan evaporation) and species P/E_p can be analyzed to find relative dominance of fixed effects vs plasticity and to demonstrate how this dominance affects reciprocal transplants. Here, we used $\dot{K}_x$ as an example trait. **A**: Contours from the regression fit to $\dot{K}_x$ observations, as in Fig. 3. Arrows show directions of steepest ascent. Arrow colors indicate dominance of fixed effect (black) or plasticity (red). The 1:1 line marks plants at their native P/E_p. Points indicate P/E_p combinations of reciprocal transplants shown in panel F. **B-D**: Hypothetical contour plots with either fixed effects only, plasticity only, or balanced effects of both. These hypothetical plots use contours in the form of Eqn. 5 and predict the same values at white and purple points as in A. **E**: Observed directions of steepest ascent have a distribution, whose mean lies at 60.2° or 0.67 of the span between fully plastic and fully fixed (vertical black line). Along the 1:1 line in panel A, all angles are 58.3° (0.65 fixed; vertical dashed line). **F**: If hypothetical species in their native environments (white and purple points) were reciprocally transplanted (red and blue points), contour plots in panels **A-D** predict their new $\dot{K}_x$ values. Observed contours predict $\dot{K}_x$ close to predictions of the balanced scenario, but shifted towards fixed-dominant.
